# Analgesic efficacy and safety of erector spinae plane block versus serratus anterior plane block in breast surgery—a meta-analysis and systematic review of randomized controlled trials

**DOI:** 10.1186/s44158-024-00218-7

**Published:** 2024-12-18

**Authors:** Samiullah Shaikh, Umm E Salma Shabbar Banatwala, Paranshi Desai, Muhammad Arham Khan, Rimsha Bint-e-Hina, Sidra Samad, Muhammad Hamza Sikandari, Ali Nawaz, Rana Ijaz, Shayan Asmat, Abeer Fatima, Harim Mirza, Noor Mahal Azam, Qurat Ul Ain Muhammad, Satesh Kumar, Mahima Khatri

**Affiliations:** 1 Liaquat University of Medical and Health Sciences, Jamshoro, Pakistan; 2 Dow University of Health Sciences, Karachi, Pakistan; 3 Bukovinian State Medical University, Chernivtsi, Ukraine; 4 Shaheed Mohtarma Benazir Bhutto Medical College Lyari, Karachi, Pakistan; 5 Allama Iqbal Medical College, Lahore, Pakistan; 6 Services Institute of Medical Sciences(SIMS), Lahore, Pakistan; 7 Jinnah Hospital, Lahore, Pakistan; 8 Rawalpindi Medical University, Rawalpindi, Pakistan

**Keywords:** ESPB, SAPB, Postoperative pain, Regional anesthesia, Breast cancer surgery

## Abstract

**Background:**

Mastectomy and breast-conserving surgery are key interventions for breast cancer, a leading cause of cancer-related mortality in women. Many undergoing breast surgery experience postoperative pain compromising their functionality and quality of life. While multiple pain management strategies are available, evidence comparing the erector spinae (ESPB) and serratus anterior plane blocks (SAPB) for improving post-surgical pain management in breast cancer surgery patients is limited. Therefore, we investigated the efficacy and safety of these two regional anesthesia techniques.

**Methods:**

After PROSPERO registration, we systematically searched PubMed, Google Scholar, and Cochrane Library until May 2024. Risk ratios (RR) were calculated for dichotomous outcomes and standard mean differences (SMD) or mean differences (MD) were computed for continuous data. RevMan Review Manager 5.4.1 was used for the data analysis and generation of forest plots as well as funnel plots. The Cochrane Risk of Bias tool 2.0 (18) and Grades of Recommendation, Assessment, Development, and Evaluation (GRADE) guidelines were used to appraise and evaluate the evidence (19).

**Results:**

A total of 9 randomized control trials enrolling 550 patients were included. Static pain scores at 0, 6, 8, 12, and 24 h after surgery, dynamic pain scores computed at 0, 8, 12, and 24 h after surgery and area under the curve (AUC) static pain score at all time points between 0 and 24 h (SMD (HKSJ 95% CI) − 0.27 [− 0.99, 0.45]) did not significantly vary with either plane block. Postoperative morphine consumption in the first 24 h and the number of patients requesting analgesia were significantly greater in those receiving SAPB [MD: − 1.41 (95% C.I. − 2.70, − 0.13), *p* = 0.03] and [RR: 1.28 (95% C.I. 1.00, 1.63), *p* = 0.05], respectively. The time to first postoperative analgesic use was significantly greater among those administered ESPB [MD: 1.55 h, (95% C.I. 1.02, 2.09), *p* < 0.01]. Patient satisfaction scores and the incidence of nausea and vomiting were similar across both groups.

**Conclusions:**

While pain scores with either block are comparable, ESPB reduces postoperative morphine consumption and may be the favorable option in breast cancer patients undergoing surgery.

**Supplementary Information:**

The online version contains supplementary material available at 10.1186/s44158-024-00218-7.

## Introduction

Mastectomy and breast-conserving surgery are pivotal in the management of breast cancer, a disease that ranks among the leading causes of cancer-related mortality in women globally [1, 2]. Projections indicate a 31% surge in breast cancer incidence by the year 2040 compared to 2020 Figs. [3]. A significant proportion of women, ranging from 25 to 80%, grapple with post-surgical pain following breast cancer surgery, leading to compromised functionality and diminished quality of life [4–6]. Such findings underscore a pressing imperative for better pain management strategies.

Direct tissue trauma, compounded by inflammatory processes at the surgical site, serves as a primary instigator of acute postoperative pain [7]. Conversely, individuals undergoing breast cancer surgery may also contend with chronic pain, encompassing phenomena such as phantom breast pain, intercostobrachial neuralgia, and pain stemming from nerve injury or neuroma development [8]. While consensus on optimal pain management strategies remains elusive, expert counsel advocates for preemptive oral analgesia, judicious use of nerve blocks (pre-, intra-, or postoperative as appropriate), and prudent restriction of opioid medications [9]. Notable nerve block techniques include the thoracic paravertebral, erector spinae, pectoralis, and serratus anterior plane blocks. While the thoracic epidural and paravertebral blocks are not appropriate for minimally invasive surgery due to associated complications, ultrasound-guided thoracic blocks such as the pectoral nerve, serratus anterior plane, and erector spinae plane blocks are gaining traction due to their efficacy and safety profiles [10].

The serratus anterior plane block (SAPB) selectively targets the lateral cutaneous branches of thoracic intercostal nerves, eliciting paresthesia across T2 to T9 dermatomes. Conversely, the erector spinae plane block (ESPB), a paraspinal fascial plane blockade, achieves a comprehensive sensory blockade by impeding the dorsal and ventral rami of thoracic spinal nerves. Its distinctive spread affords abdominal visceral analgesia as well, rendering it a promising option [11, 12]. Recently, ESPB has garnered more interest due to its use in various surgeries. Multiple meta-analyses published have investigated ESPB in providing postoperative pain relief in a multitude of procedures ranging from sternotomies, spinal surgeries, and laparoscopic abdominal procedures shedding light on its diversified use [13, 14, 15].

Although previous systematic reviews and meta-analyses have delved into comparing the ESPB with the pectoralis nerve block and paravertebral block [16], or the SAPB with the thoracic paravertebral block [17], a notable gap exists in evidence synthesis concerning the ESPB versus the SAPB. Given the proliferation of trials directly comparing the efficacy and safety profiles of these two plane blocks, consolidating this evidence is imperative to discern the superior option for breast surgeries. Thus, this systematic review and meta-analysis seeks to investigate and compare the analgesic efficacy and safety of the erector spinae plane block versus the serratus anterior plane block in the context of breast surgery.

## Methods

This systematic review and meta-analysis was conducted following the preferred reporting items for Systematic Reviews and Meta-Analyses (PRISMA) guidelines [18]. The protocol for this review was registered in the PROSPERO (International Prospective Register for Systematic Reviews) database with ID CRD42024507382.

### Eligibility criteria

Randomized controlled trials (RCTs) that met the following criteria were included: (1) studies assessing the use of erector spinae plane block (ESPB) as the intervention and serratus anterior plane block (SAPB) as the comparator, (2) patients undergoing breast cancer surgery (radical mastectomy or modified radical mastectomy), (3) assessing outcomes related to analgesic efficacy and safety, (4) area under the curve (AUC) pain score at static between 0 and 24 h, (5) postoperative morphine or morphine equivalent (mg) consumption in first 24 h postoperatively, and (6) studies providing full-text access, either in English or any other language. Moreover, articles that did not provide the data necessary for calculating a mean difference or standard mean difference (MD or SMD) and a 95% confidence interval (CI) were excluded.

### Literature search strategy

A systematic literature search was conducted on electronic databases and included PubMed, Google Scholar, and Cochrane Library from inception through May 2024. The keywords used to retrieve all pertinent publications were: “erector spinae plane block,” “serratus anterior plane block,” “breast surgery,” “postoperative analgesia,” and “pain management.” The detailed search technique is provided in the online supplementary appendix A. Furthermore, the bibliography of potentially eligible articles was examined for relevant studies.

### Study selection process

Two independent reviewers [P.D. and S.S.] screened titles and abstracts of retrieved studies, and full texts of potentially eligible studies were assessed for final inclusion. Zotero was used to store references and remove any duplicate studies. Discrepancies were resolved through discussion or consultation with a third reviewer [U.S.S.B].

### Data extraction

Data extraction forms were created on Google Sheets by the data extraction team [S.S and R.B.H]. Each team member independently extracted data. Extracted data were verified by a third reviewer [N.M.A]. Relevant data extracted from included studies are as follows: (1) study characteristics: first author name, publication year, and study design; (2) patient demographics: total number of participants, mean age, gender, and BMI; (3) intervention details: type of surgery, type of block performed (ESPB or SAPB), technique used, and analgesic regimen used; (4) outcome measures: primary outcomes included AUC postoperative pain scores between 0 and 24 h and postoperative opioid consumption in first 24 h, and secondary outcomes included postoperative static and dynamic pain scores at 0, 6, 8, 12, and 24 h, time to first postoperative analgesic request, patient satisfaction score, and incidence of adverse events (e.g., nausea, vomiting). For continuous outcome data, mean and standard deviation were extracted. Standardized statistical conversions were made if the data were reported as median and interquartile range (IQR) and conversion was done on online calculator [19, 20]. Dichotomous data were extracted in events/total format. Graphical data were extracted using the Plot digitizer online application [21].

### Primary and secondary outcomes

The co-primary outcomes evaluated in this systematic review and meta-analysis were the area under the curve of postoperative pain scores static between 0 and 24 h (AUC pain score at static between 0 and 24 h) and postoperative oral morphine or morphine equivalent consumption in 24 h. The secondary outcomes were postoperative pain scores static at 0, 6, 8, 12, and 24 h and dynamic at 0, 8, 12, and 24 h, time to the first dose of postoperative opioids, number of patients requested opioids postoperatively, patient satisfaction score, and incidence of vomiting and nausea.

### Quality assessment and risk of bias

To evaluate the methodological quality of the included RCTs, the Cochrane Collaboration Risk-of-Bias tool 2.0 [22] was employed. This tool comprises five domains, namely, bias arising from randomization, bias due to deviations from planned interventions, bias due to missing outcome data, bias in the measurement of the outcome, and bias of selective reporting. Two independent reviewers [R.I and A.N] assessed each trial’s methodology and assigned a risk of bias rating as low, unclear, or high based on predetermined criteria. Any discrepancies between the reviewers were resolved through reevaluation by a third reviewer [S.S.].

For the assessment of the overall strength of evidence, the GRADE guidelines [23] were utilized. These guidelines classify the strength of evidence into four levels: high-quality (⊕ ⊕ ⊕ ⊕), moderate-quality (⊕ ⊕ ⊕ ⊖), low-quality (⊕ ⊕ ⊖ ⊖), and very-low-quality (⊕ ⊖ ⊖ ⊖) evidence.

### Measurement of outcomes

Postoperative pain scores were assessed both at rest (static) and during movement (dynamic) at 0, 6, 8, 12, and 24 h after the surgery. The pain score data were transformed into an equivalent score on a 0–10-cm visual analog scale (VAS), with 0 cm corresponding to no pain and 10 cm corresponding to the worst experienced pain. Additionally, the doses of various postoperative opioids consumed within 24 h were converted into equivalent doses of oral morphine in milligrams using a standardized converter [24]. All time-to-event data was converted into hours for uniformity and ease of comparison.

### AUC analysis

AUC of rest pain scores was calculated for each trial between 0 and 24 h time interval using the trapezoid method [25]. Pooling the trials was then done using the HKSJ method for random effects [26].

### Interpretation

We looked at our main results to see if they were important for patients in terms of minimal clinically important difference (MCID) [27]. For the “postoperative pain scores (static and dynamic),” we considered it meaningful if the average pain score [28] on the visual analog scale (VAS) decreased by more than 1.1 cm. For the “24-h postoperative oral morphine (mg) equivalent consumption,” we considered it significant [28] if the average amount of morphine used was reduced by more than 30 mg.

### Statistical analysis

For the meta-analysis, Review Manager 5.4.1 software was utilized to generate funnel and forest plots. The following methods were employed for calculating effect sizes and conducting statistical tests: The generic-inverse variance method with a random-effects model was employed to calculate the standard mean difference (SMD) for continuous variables related to postoperative pain scores due to the diversity of scales used and mean difference (MD) for the rest of the continuous variables along with the corresponding 95% confidence interval (CI). For dichotomous variables, the Mantel–Haenszel method with a random-effects model was utilized to calculate the risk ratio (RR). The threshold for statistical significance was set at *p* < 0.05.

The results of the pooled studies were visually represented using forest plots, which provide a graphical display of the effect sizes and their confidence intervals. Funnel plots were constructed to assess publication bias of the outcomes via visual inspection. The degree of inconsistency among the included studies was assessed using Higgins’ *I*^2^ test, which quantifies the percentage of total variation across studies due to heterogeneity rather than chance. The degree of heterogeneity was categorized as follows: Low heterogeneity:* I*^2^ < 25%, moderate heterogeneity: *I*^2^ 25–75%, and high heterogeneity: *I*^2^ > 75%. Moderate and high levels of heterogeneity prompted exploration into the potential causes of heterogeneity as outlined in the literature [29].

### Methods to explore causes of heterogeneity

To understand the causes of differences in results, we employed following analytical approaches. Firstly, we conducted a sensitivity analysis where we sequentially excluded studies based on (1) the usage or non-usage of alprazolam and midazolam as adjuncts on the day of surgery and (2) the use of any local anesthetic (LA) other than the most commonly used LA, i.e., bupivacaine. This analysis helped us assess how the results changed when specific studies were removed from the analysis, providing insights into the reliability of our findings. Additionally, we performed a subgroup analysis for our two primary outcomes by dividing the studies into two subgroups, i.e., “Bupivacaine vs others” before and after excluding the studies sequentially. The usage of benzodiazepine as adjuncts on the day of surgery and LA other than bupivacaine were identified as potential sources of high heterogeneity.

## Results

### Study selection

**Fig. 1:**
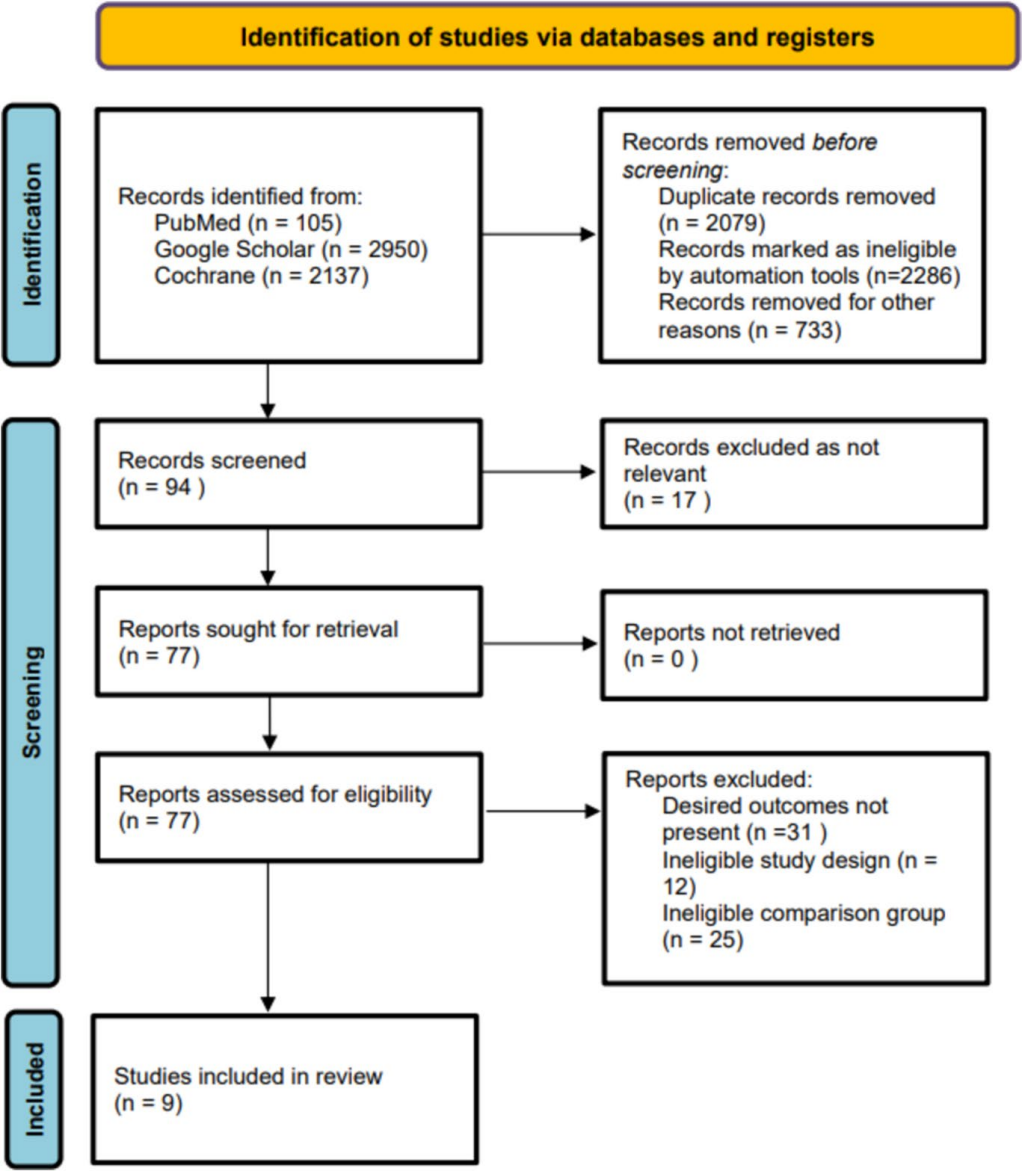
PRISMA flowchart. PRISMA Preferred Reporting Items for Systematic Reviews and Meta-Analyses

An extensive literature search yielded a total of 5192 results. Considering the study eligibility criteria, 94 articles were subjected to screening after reviewing the titles and abstract. The remaining records were scrutinized based on full-text, and after ruling out 85 studies due to ineligible study design and comparison group, 9 RCTs [30–38] were included in this meta-analysis (Fig. 1).

### Study and patient characteristics

A total of nine RCTs were included in this meta-analysis. These studies were conducted in various countries, such as Egypt, China, and India, from 2019 to 2024. The included number of patients was 550 as demonstrated by Table 1. The number of participants in the studies ranged from 40 to 100. The population assessed in a number of these studies is females undergoing radical mastectomy [37] and modified radical mastectomy (MRM) [30, 31, 33–37] (Table 1). Four studies [32–35] used 0.25%, 0.375 and 0.5% ropivacaine, four studies [31, 36–38] used 0.25% bupivacaine and one study [30] used 2% articaine. The baseline characteristics of studies and anesthesia are specified in Tables 1 and 2.

**Table 1:** Baseline characteristics of included RCTs

Author, year	Total number of patients	Number of patients in each group		Age		Gender (M/F)		BMI		Physical status ASA I/II/III		Type of surgery
		**ESPB**	**SAPB**	**ESPB**	**SAPB**	**ESPB**	**SAPB**	**ESPB**	**SAPB**	**ESPB**	**SAPB**	
Eldemrdash 2019 [30]	50	25	25	55 (2.9)	50.2 (7.8)	F	F	26.2 (1.8)	23.5 (3.2)	14/11	12/13	MRM
Wang 2019 [32]	100	50	50	52 (7)	49 (7)	F	F	N/A	N/A	N/A	N/A	Radical mastectomy
Elsabeeny 2020 [31]	50	25	25	51.80 (9.07)	52.44 (8.70)	F	F	28.05 (2.4)	27.30 (2.5)	N/A	N/A	MRM
Shrivastava 2021 [38]	50	25	25	45.41 (11.22)	44.40 (10.45)	F	F	160 (30)	161 (20)	N/A	N/A	Unspecified
Jiang 2021 [33]	60	30	30	54.73 (13.60)	52.10 (11.50)	F	F	23.40 (3.0)	23.52 (3.1)	13/17	18/12	MRM
Sagar 2022 [37]	40	20	20	53.95 (4.796)	53.90 (4.064)	F	F	25.53 (2.5)	24.89 (3.21)	N/A	N/A	MRM
Ahuja 2022 [35]	80	40	40	49.6 (11.5)	45.9 (10.0)	F	F	26.5 (4.4)	24.89 (3.6)	15/24/1	23/17/0	MRM
Nyima 2023 [34]	80	40	40	50.38 (11.24)	53.08 (14.18)	F	F	N/A	N/A	20/20	18/22	MRM
Bedewy 2024 [36]	40	20	20	47 (7.54)	45.65 (5.40)	F	F	N/A	N/A	N/A	N/A	MRM

*ESPB* erector spinae plane block, *SAPB* serratus anterior plane block, *N/A* not applicable, *BMI* body mass index, *ASA* American Standards Association

**Table 2:** Details of block procedure and analgesic regimens in the included RCTs

Author/year	Preincisional analgesia and anti-emetics	Block timing	ESPB local anesthetic bolus	SAPB local anesthetic bolus	Localization	Supplemental and postoperative analgesia
Eldemrdash 2019 [30]	One gram of paracetamol was administered IV after induction of anesthesia	Preoperative after (GA)	20 ml of 2% articaine	20 ml of 2% articaine	USG	One gram paracetamol was administered IV every 8 h
Wang 2019 [32]	1 g/kg dexmedetomidine was pumped (the pump was completed within 10 min)	Preoperative before (GA)	20 ml of 0.375% ropivacaine	20 ml of 0.375% ropivacaine	USG	When the VAS score was > 4 points, 50 mg of flurbiprofen axetil was added for analgesia. If the VAS score was still > 4 points after 30 min, sufentanil 5 g was given for each rescue analgesia, the dosage and times of flurbiprofen axetil and sufentanil rescue were recorded
Elsabeeny 2020 [31]	Patients were pre-medicated with midazolam (2 mg IV) and metoclopramide 0.1 mg/kg	Preoperative after (GA)	25 ml of 0.25% bupivacaine	25 ml of 0.25% bupivacaine	USG	Ketorolac when the VAS score was < 4
Shrivastava 2021 [38]	Intravenous ondansetron 4 mg and dexamethasone 8 mg	Preoperative after (GA)	25 ml of 0.25% bupivacaine	25 ml of 0.25% bupivacaine	USG	When VAS score was ≥ 3, intravenous diclofenac 75 mg was given. If VAS was still ≥ 3, tramadol 50 mg was given till VAS < 3
Jiang 2021 [33]	N/A	Preoperative after (GA)	20 ml of 0.5% ropivacaine	20 ml of 0.5% ropivacaine	USG	Within 24 h after the operation, the patients received an intravenous injection of tramadol 1–2 mg/kg for pain relief until the NRS pain score was < 3, and again if the postoperative NRS was greater than 3 points, tramadol 1–2 mg/kg was administered int ravenously, and then the pain was evaluated after 30 min
Ahuja 2022 [35]	Intravenous dexamethasone (0.1 mg kg − 1) and paracetamol (15 mg kg − 1) were administered immediately after induction	Preoperative before (GA)	30 ml of 0.25% ropivacaine	30 ml of 0.25% ropivacaine	USG	Intravenous infusion of paracetamol of 15 mg kg − 1 immediately after induction and repeated every 8 h for the first 24 h. On assessment, if NRS was ≥ 3 at rest or arm abduction, first rescue analgesia, intravenous diclofenac sodium (1.5 mg kg 1) was administered; not repeated within 12 h of the first dose. Patients were reassessed after 30 min; if NRS ≥ 3 persisted, a second rescue analgesic intravenous tramadol hydrochloride (1 mg kg 1) was administered
Nyima 2021 [34]	Oral ranitidine 150 and 0.25 mg alprazolam at night before surgery and at 6AM on the day of surgery	Preoperative after (GA)	20 ml of 0.25% ropivacaine	20 ml of 0.25% ropivacaine	USG	1 g of paracetamol was administered to patients in both the study groups in case the VAS score > 4. Rescue analgesia with 1 g of paracetamol was administered to patients in both the study groups in case the VAS score > 4
Sagar 2022 [37]	Ranitidine 150 mg, tablet metoclopramide 10 mg and alprazolam 0.25 mg peroral on the night before and the day of the scheduled surgery as premedication	Preoperative after (GA)	24 ml of 0.25% bupivacaine	24 ml of 0.25% bupivacaine	USG	If the NRS was ≥ 4, rescue analgesics were administered. Tramadol 1–2 mg kg-1 was given IV as a first-line drug. If the pain persisted, paracetamol 15 mg kg-1 was administered IV
Bedewy 2024 [36]	Intravenous dose of midazolam ranging from 0.01 to 0.02 mg/kg, administered 30 min before the operation	Preoperative after (GA)	20 ml of 0.25% of bupivacaine	20 ml of 0.25% of bupivacaine	USG	IV morphine at 0.05 mg/kg/dose was administered when NRS score > 4

*GA* general anesthesia, *ESPB* erector spinae plane block, *SAPB* serratus anterior plane block, *LA* local anesthesia, *USG* ultrasound-guided, *IV* intravenous, *NRS* numerical rating scale, *SAM* serratus anterior muscle, *DEX* dexamethsone, *VAS* visual analog scale

### Risk of bias

**Fig. 2:**
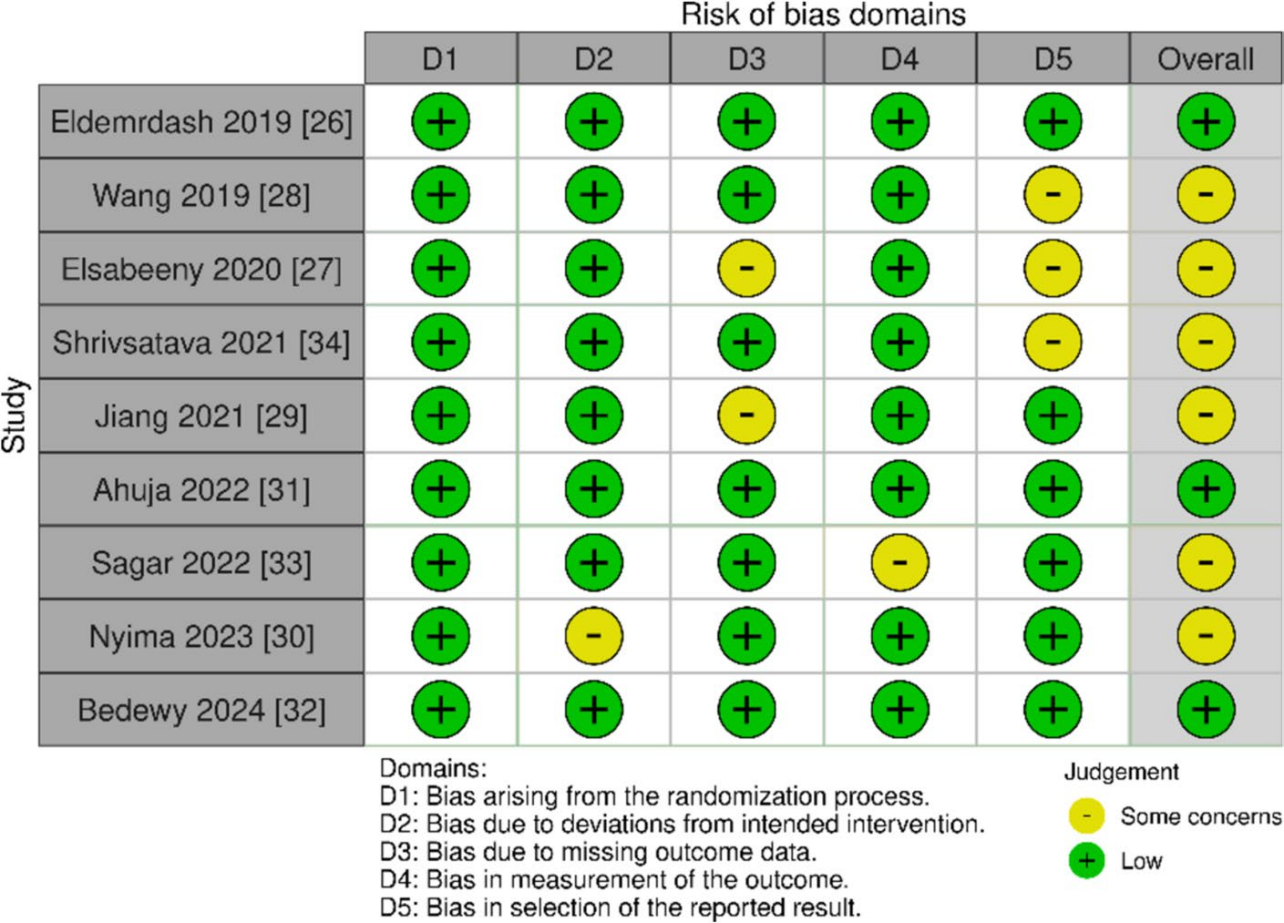
Risk of bias assessment for the included trials using Cochrane risk of bias tool 2.0

All nine studies [30–38] adequately described the random sequence generation methods and reported using allocation concealment to reduce bias. Eight studies showed low risk for detection bias [30–36, 38] and performance bias [30–33, 35–38]. Seven studies [30, 32, 34–38] explicitly reported low risk for attrition bias. Six studies [30, 33–37] were low risk for reporting bias (Fig. 2).

### Outcomes

Outcomes are represented in a tabulated form in online supplementary appendix B.

### Primary outcomes

#### AUC pain score at static between 0 and 24 h

**Fig. 3:**
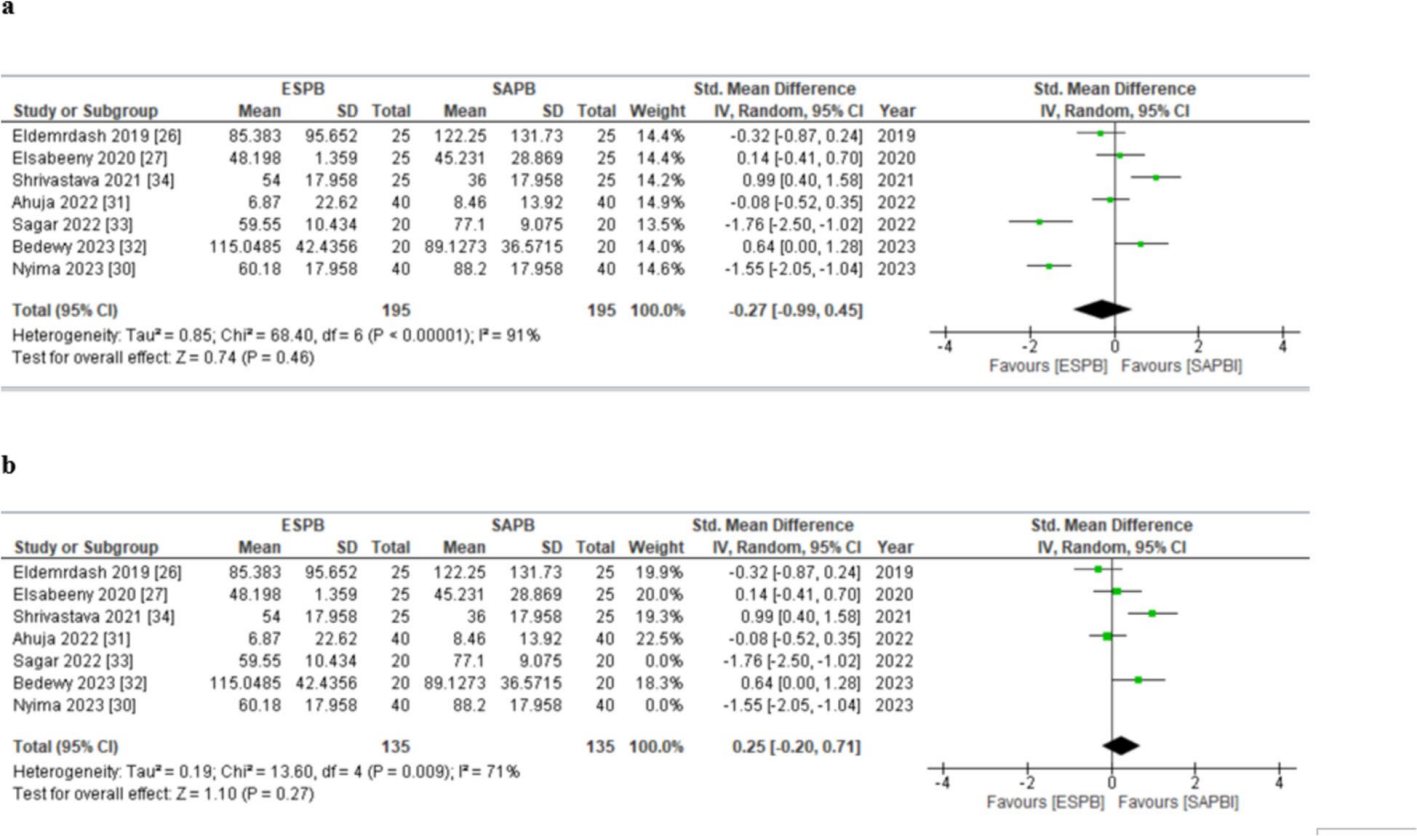
**a** Forest plot of AUC postoperative pain scores (static) between 0 and 24 h. The SMD estimates for each study are represented by squares, and the lines passing through them represent 95% CI. The diamond represents the overall pooled estimate. **b** Postoperative pain scores (static) at 24 h (forest plot of sensitivity analysis). SMD standard mean difference, CI confidence interval, IV inverse variance, SD standard deviation, ESPB erector spinae plane block, SAPB serratus anterior plane block

Seven studies [30, 31, 34–38] inclusive of 390 patients (ESPB: 195, SAPB: 195) reported rest pain scores at all time points between 0 and 24 h. The pooled analysis showed that AUC pain score in patients receiving ESPB and SAPB did not significantly vary (SMD (HKSJ 95% CI) − 0.27 [− 0.99, 0.45], *p* = 0.46, *I*^2^ = 91%) (Fig. 3a). None of the SMDs reached the threshold of minimal clinically important difference (MCID). This analysis was characterized by substantial inconsistency (*I*^2^ = 91%), but our results were robust to sensitivity analysis; the exclusion of studies [34, 37] on the basis of use of alprazolam in the morning on the day of surgery that synergistically enhances postoperative analgesic effect reduced the heterogeneity to 71% (SMD (HKSJ 95% CI) = 0.25 (–0.20 to 0.71) (*p* = 0.27, *I*^2^ = 71%) (Fig. 3b). Visually, the funnel plot appeared symmetrical (online supplemental appendix C), indicating an absence of publication bias. Overall, the GRADE strength of evidence was moderate (Table 2).

**Table 3:** Evidence profile for patients receiving erector spinae vs serratus anterior plane block in breast cancer surgeries

Outcomes	No. of participants (studies)	Publication bias	Mean difference or RR (95%CI)	Indirectness	Strength or certainty of the evidence	Inconsistency	Limitations
Post operative morphine or morphine equivalent (mg) consumption in the first 24 h postoperatively	550 (9)	Not detected	MD − 1.41(− 27, − 0.13)	Not detected	⨁⨁⨁◯ Moderate	High test for inconsistency (but resolved on subgroup analysis) (*I*^2^ = 94%)	No serious limitations
AUC postoperative pain score at 0–24 h (static)	390 (7)	Not detected	SMD − 0.27 (− 0.99, 0.45)	Not detected	⨁⨁⨁◯ Moderate	High test for inconsistency (resolved on SA) (*I*^2^ = 91%)	No serious limitations
Post operative pain scores (static) at 24 h	550 (9)	Publication bias strongly suspected	SMD − 0.13 (− 1, 0.74)	Not detected	⨁⨁⨁◯ Moderate	High test for inconsistency (*I*^2^ = 95%) (resolved on SA)	No serious limitations
Post operative pain scores (dynamic) at 24 h	240 (3)	Publication bias strongly suspected	SMD − 0.1 (− 1, 0.74)	Not detected	⨁⨁⨁◯ Moderate	Low test for inconsistency (*I*^2^ = 0%)	No serious limitations
No. of patients requested analgesia in the first 24 h postoperatively	260 (4)	Not detected	1.28 [1.00,1.63]	Not detected	⨁⨁⨁⨁ High	Low test for inconsistency (*I*^2^ = 0%)	No serious limitations
Time to first postoperative analgesia use (h)	360 (6)	Not detected	MD 1.55 (1.02, 2.09)	Not detected	⨁⨁⨁⨁ High	High test for inconsistency (*I*^2^ = 79%) (resolved on SA)	No serious limitations
Patient satisfaction score	140 (2)	Not detected	− 0.33 [− 0.66, − 0.01]	Not detected	⨁⨁⨁⨁ High	Low test for inconsistency (*I*^2^ = 0%)	No serious limitations

*RR* risk ratio, *MD* mean difference, *SMD* standardized mean difference, *AUC* area under curve, *CI* confidence interval, *SA* sensitivity analysis

#### Postoperative morphine or morphine equivalent (mg) consumption in the first 24 h postoperatively

**Fig. 4:**
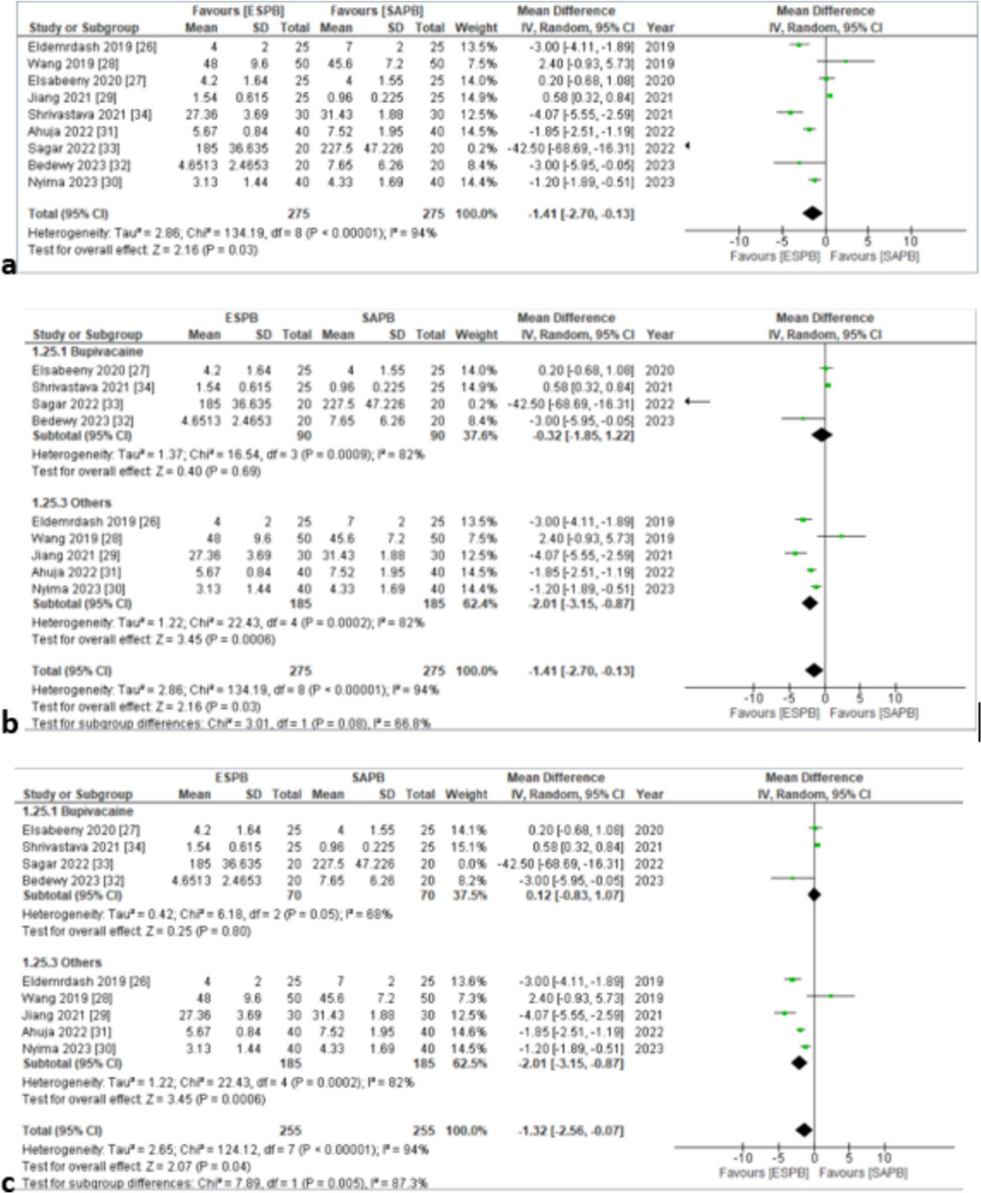
**a** Forest plot of 24-h postoperative oral morphine (mg) equivalent consumption. The MD estimates for each study are represented by squares and the lines passing through them represent 95% CI. The diamond represents the overall pooled estimate. **b** 24-h postoperative oral morphine (mg) equivalent consumption (forest plot for subgroup analysis). **c** 24-h postoperative oral morphine (mg) equivalent consumption (forest plot for sensitivity analysis in group bupivacaine). MD mean difference, CI confidence interval, IV inverse variance, SD standard deviation, ESPB erector spinae plane block, SAPB serratus anterior plane block

Nine studies [30–38] inclusive of 550 (ESPB: 275, SAPB: 275) reported morphine or morphine equivalent (mg) consumption in the first 24 h postoperatively. The pooled analysis of equivalent doses of oral morphine in milligrams revealed that the dose of morphine consumption was lower in the ESPB group as compared to the SAPB group and the results were statistically significant (MD = − 1.41 [− 2.7, − 0.13], *p* < 0.03, *I*^2^ = 94%) (Fig. 4a). Although statistically significant, the results were clinically insignificant based on the set criteria. This analysis was characterized by substantial inconsistency, but sensitivity analysis by excluding the study [38] based on the lowest time taken for surgery and unspecified surgery type failed to resolve inconsistency for this outcome (*I*^2^ = 86%). Furthermore, subgroup analysis was performed by dividing the studies based on LA modality, i.e., “Bupivacaine” and “Others” (Fig. 4b). There was substantial inconsistency, for which sensitivity analysis was done. On sensitivity analysis, the exclusion of the study [37] based on the use of alprazolam on the day of surgery that synergistically enhances postoperative analgesic strength reduced heterogeneity in the subgroup “Bupivacaine” to 68% (Fig. 4c). There was a symmetrical appearance on visual inspection of the funnel plot (online supplemental appendix C); thus, the GRADE strength of evidence was moderate (Table 2).

### Secondary outcomes

#### Postoperative pain score at 0, 6, 8, 12, and 24 h (static)

**Fig. 5:**
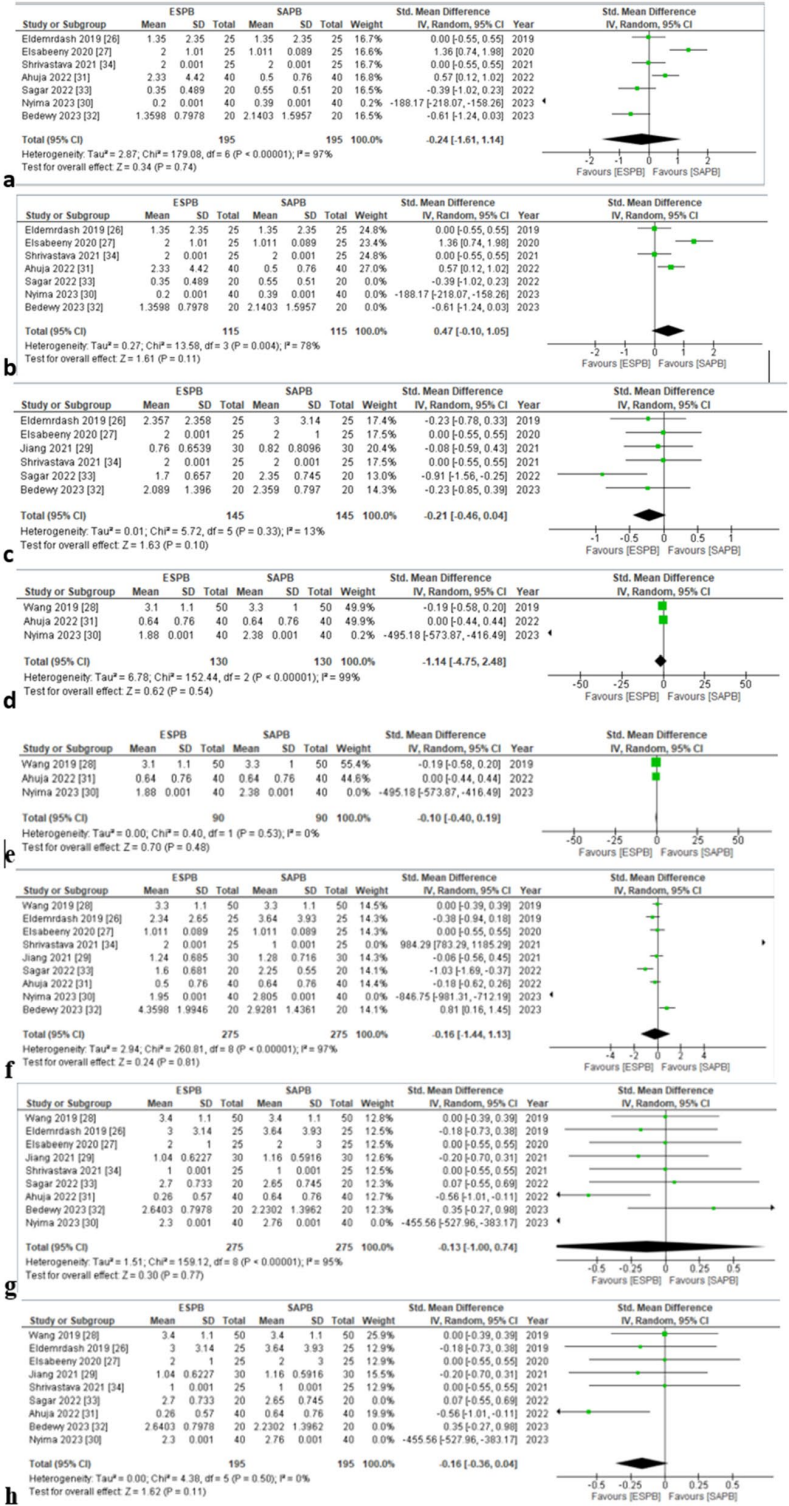
**a** Postoperative pain scores (static) at 0 h. **b** Postoperative pain scores (static) at 0 h (forest plot for sensitivity analysis). **c** Postoperative pain scores (static) at 6 h. **d** Postoperative pain scores (static) at 8 h. **e** Postoperative pain scores (static) at 8 h (forest plot for sensitivity analysis). f Postoperative pain scores (static) at 12 h. g Postoperative pain scores (static) at 24 h. h Postoperative pain scores (static) at 24 h (forest plot for sensitivity analysis). SMD standard mean difference, CI confidence interval, IV inverse variance, SD standard deviation, ESPB erector spinae plane block, SAPB serratus anterior plane block

Seven studies [30, 31, 34–38] reported postoperative pain scores at 0 h (static). No significant differences were observed between the two groups for this outcome (SMD = − 0.24 [− 1.61,1.14], *p* = 0.74, *I*^2^ = 97%) (Fig. 5a). High in-study heterogeneity was seen. Sensitivity analysis was performed by removing studies based on usage of benzodiazepine, i.e., alprazolam 0.25 mg [34, 37] and midazolam 0.01–0.02/mg/kg [36] on the day of surgery, (SMD = 0.47 [− 0.10,1.05], *P* = 0.11, *I*^2^ = 78%) heterogeneity reduced to 78% (Fig. 5b). Visually, the funnel plot appeared asymmetrical (online supplemental appendix C).

Postoperative pain score at 6 h (static) was assessed by six studies [30, 31, 33, 36–38]. The pooled analysis showed that pain score at 6 h did not vary significantly with either plane block (SMD = –0.21 [–0.46, 0.04], *p* = 0.10, *I*^2^ = 13%) (Fig. 5c). Heterogeneity was found to be low. There was an asymmetrical appearance on the visual inspection of the funnel plot (online supplemental appendix C).

Three studies [32, 34, 35] evaluated postoperative pain score at 8 h (static). According to the pooled analysis, none of the plane blocks were superior to the other (SMD = –1.14 [–4.75, 2.48], *p* = 0.54, *I*^2^ = 99%) (Fig. 5d). Heterogeneity was found to be high. In order to reduce heterogeneity, sensitivity analysis was done by removing a study based on the usage of alprazolam 0.25 mg [34] (SMD = –0.10 [–0.40,0.19], *p* = 0.48, *I*^2^ = 0%) (Fig. 5e). The funnel plot observed an asymmetrical pattern (online supplemental appendix C).

Postoperative pain score at 12 h (static) was assessed by nine studies [30–38]. The pooled analysis revealed insignificant differences among both the plane blocks (SMD = –0.16 [–1.44, 1.13], *P* = 0.81, *I*^2^ = 97%) (Fig. 5f). High in-study heterogeneity was observed. Sensitivity analysis by removing studies based on the usage of benzodiazepine, i.e., alprazolam 0.25 mg [34, 37] and midazolam 0.01–0.02/mg/kg [36] on the day of surgery (SMD = –0.10, [–1.13,0.94] *p* = 0.85, *I*^2^ = 95%) failed to reduce significant heterogeneity. The funnel plot demonstrated symmetry (online supplemental appendix C).

All nine studies [30–38] reported postoperative pain score at 24 h (static). Both regional blocks yielded similar postoperative pain score (static) at 24 h according to the pooled analysis (SMD = − 0.13 [− 1.00, 0.74], *p* = 0.77, *I*^2^ = 95%) (Fig. 5g). Heterogeneity was seen to be high. On conducting sensitivity analysis by removing studies based on usage of benzodiazepine, i.e., alprazolam 0.25 mg [34, 37] and midazolam 0.01–0.02/mg/kg [36] on the day of surgery (SMD = − 0.16, [− 0.36, 0.04], *p* = 0.11), heterogeneity dropped to 0% (Fig. 5h). Symmetry was observed on the funnel plot (online supplemental appendix C). Overall, the GRADE strength of evidence was low in static pain score at 24 h (Table 2).

#### Postoperative pain scores at 0, 8, 12, and 24 h (dynamic)

**Fig. 6:**
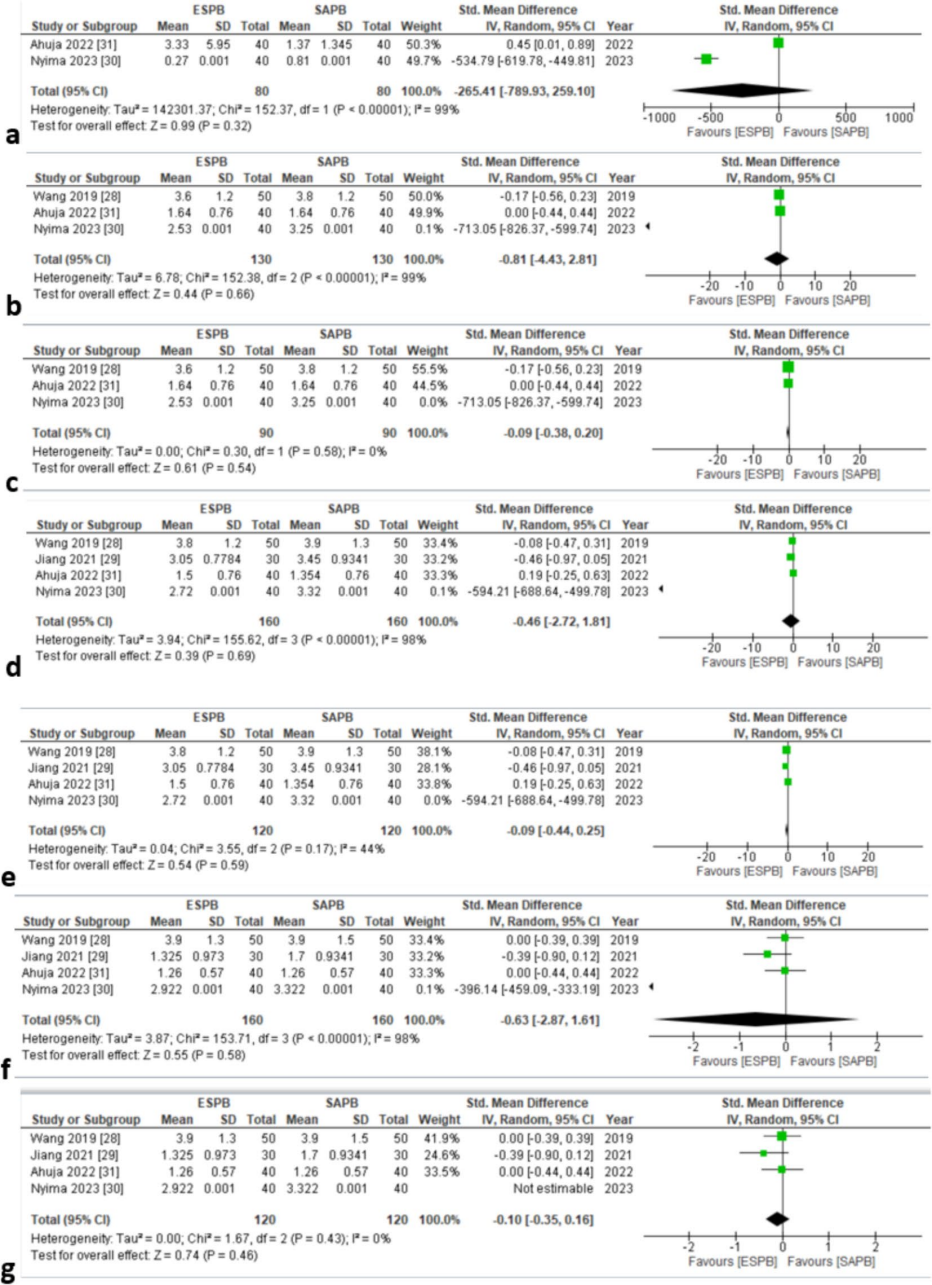
**a** Postoperative pain scores (dynamic) at 0 h. **b** Postoperative pain scores (dynamic) at 8 h. **c** Postoperative pain scores (dynamic) at 8 h (forest plot for sensitivity analysis). **d** Postoperative pain scores (dynamic) at 12 h. **e** Postoperative pain scores (dynamic) at 12 h (forest plot for sensitivity analysis). **f** Postoperative pain scores (dynamic) at 24 h. **g** Postoperative pain scores (dynamic) at 24 h (forest plot for sensitivity analysis). SMD standard mean difference, CI confidence interval, IV inverse variance, SD standard deviation, ESPB erector spinae plane block, SAPB serratus anterior plane block

Two studies [34, 35] assessed postoperative pain score at 0 h (dynamic). The pooled analysis showed insignificant results (SMD = − 265.41 [− 789.93, 259.10], *P* = 0.32, *I*^2^ = 99%) (Fig. 6a). High in-study heterogeneity was observed. The funnel plot appeared symmetrical (online supplemental appendix C), indicating the absence of publication bias.

Three studies [32, 34, 35] reported postoperative pain scores at 8 h (dynamic). Insignificant results were yielded through the pooled analysis (SMD = − 0.81 [− 4.43, 2.81], *p* = 0.66, *I*^2^ = 99%) (Fig. 6b). Heterogeneity was seen to be high. Sensitivity analysis was conducted by removing a study based on the usage of alprazolam 0.25 mg [34] (SMD = − 0.09 [− 0.38, 0.20], *p* = 0.54, *I*^2^ = 0%), and heterogeneity dropped to 0% (Fig. 6c). Visually, the funnel plot exhibited asymmetry (online supplemental appendix C).

Four studies [32–35] evaluated postoperative pain scores at 12 and 24 h (dynamic). The pooled analysis revealed insignificant differences between the two groups (SMD = − 0.46 [− 2.72, 1.81], *p* = 0.69, *I*^2^ = 98%) (Fig. 6d) and groups (SMD = − 0.46 [− 2.72, 1.81], *p* = 0.69, *I*^2^ = 98%) (Fig. 6f). High in-study heterogeneity was seen in both outcomes. To reduce the heterogeneity, sensitivity analysis was conducted by excluding a study [34] based on the usage of alprazolam 0.25 mg (SMD = − 0.09 [− 0.44, 0.25], *p* = 0.59, *I*^2^ = 44%) (Fig. 6e) and (SMD = − 0.10 [− 0.35, 0.16], *p* = 0.46, *I*^2^ = 0%) (Fig. 6g), respectively. Both the funnel plots exhibited an asymmetrical pattern (online supplementary appendix C).

Overall, the GRADE strength of evidence was moderate for dynamic pain score outcome at 24 h (Table 2).

#### Number of patients requested analgesia in the first 24 h postoperatively

**Fig. 7:**
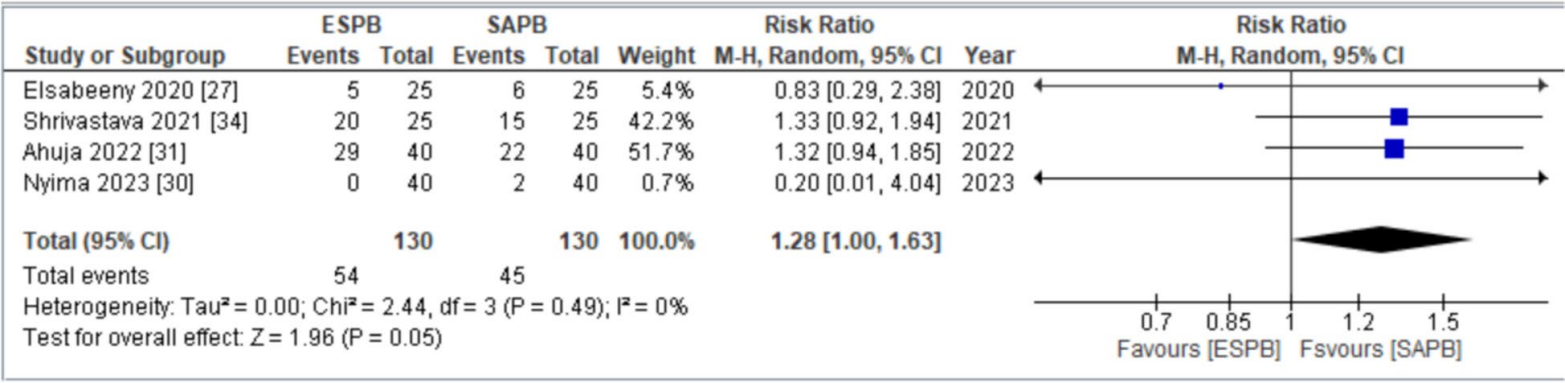
Forest plot for number of patients requested analgesia in first 24 h postoperatively. RR relative risk, CI confidence interval, M–H Mantel–Haenszel, SD standard deviation, ESPB erector spinae plane block, SAPB serratus anterior plane block

Four studies [31, 32, 34, 38] assessed the number of patients requesting analgesics in the first 24 h postoperatively. The pooled analysis revealed that the number of patients who received SAPB showed increased demand of analgesia in the first 24 h postoperatively. Statistically significant difference was obtained between the two groups (RR = 1.28 [1.00,1.63], *p* = 0.05, *I*^2^ = 0%) (Fig. 7). Low in-study heterogeneity was observed. There was an asymmetrical appearance on visual inspection of the funnel plot (online supplemental appendix C); thus, the GRADE strength of evidence was high (Table 2).

### *Time to first postoperative analgesic use (hours)*

**Fig. 8:**
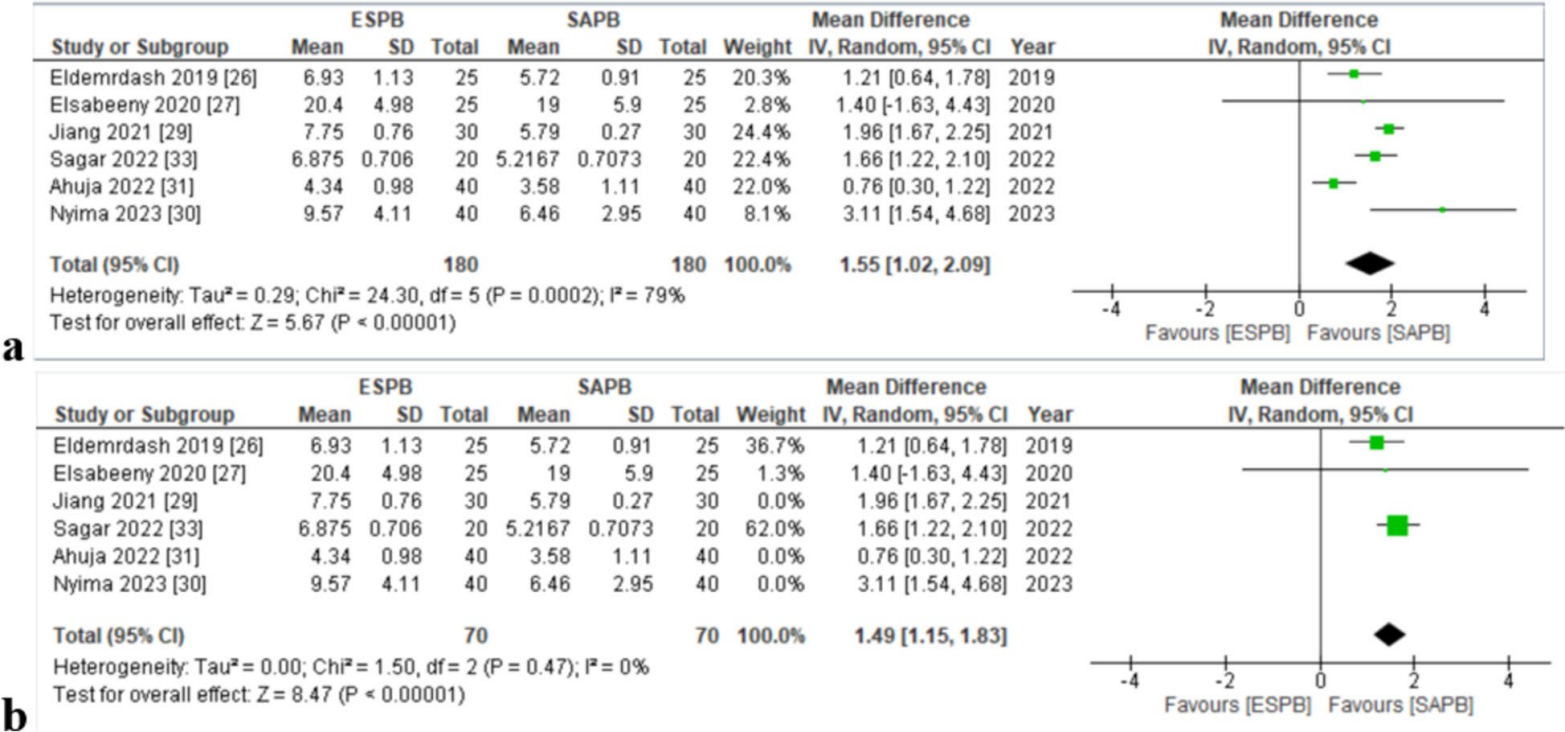
**a** Forest plot for time to request the first dose of postoperative analgesia. **b** Forest plot for time to request the first dose of postoperative analgesia (sensitivity analysis). MD mean difference, CI confidence interval, IV inverse variance, SD standard deviation, ESPB erector spinae plane block, SAPB serratus anterior plane block

Six articles [30, 31, 33–35, 37] analyzed the time to first postoperative analgesic use (hours). Significant results were obtained. The pooled analysis exhibited that the patients receiving ESPB took more time to first postoperative analgesic use (MD = 1.55, [1.02, 2.09], *p* = < 0.01, *I*^2^ = 79%) (Fig. 8a). High in-study heterogeneity was seen. On performing sensitivity analysis by removing studies based on usage of ropivacaine [33–35] and usage of alprazolam 0.25 mg on the day of surgery [34] (MD = 1.49 [1.15,1.83], *p* = < 0.01, *I*^2^ = 0%), heterogeneity dropped to 0% (8b). The funnel plot appeared symmetrical (online supplemental appendix C), indicating the absence of publication bias. Overall, the GRADE strength of evidence was high (Table 2).

#### Patient satisfaction score

**Fig. 9:**
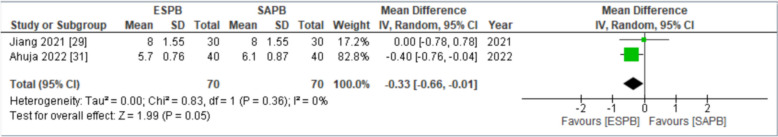
Forest plot for postoperative satisfaction score. MD mean difference, CI confidence interval, IV inverse variance, SD standard deviation, ESPB erector spinae plane block, SAPB serratus anterior plane block

It was assessed by two articles [33, 35]. Patients in both groups demonstrated similar satisfaction according to the pooled analysis (MD = − 0.33 [− 0.66, − 0.01], *p* = 0.05, *I*^2^ = 0%) (Fig. 9). No in-study heterogeneity was observed. There was a symmetrical appearance on visual inspection of the funnel plot (online supplemental appendix C). The GRADE strength of evidence was found to be high (Table 2).

#### Postoperative nausea and vomiting

**Fig. 10:**
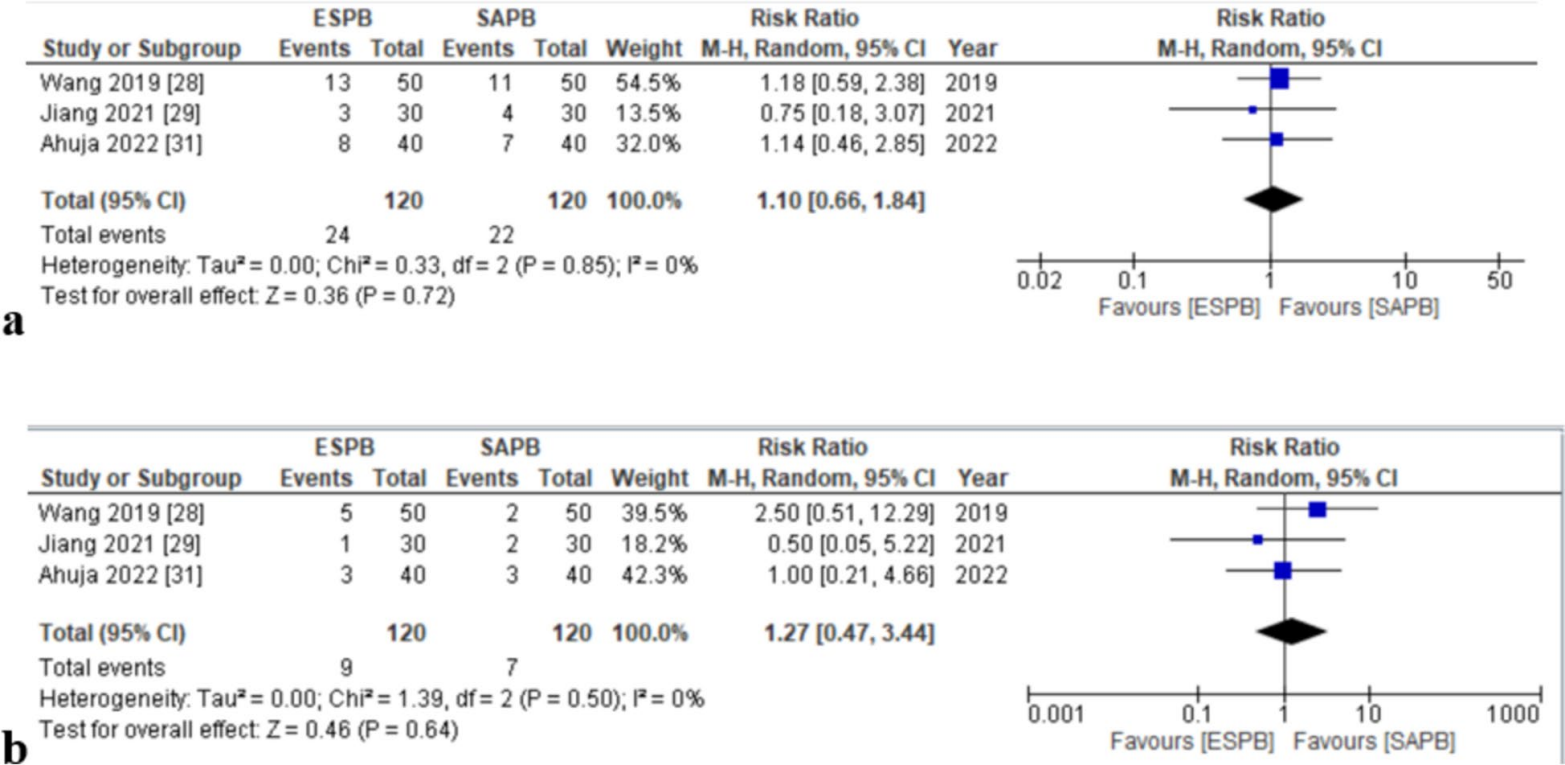
**a** Forest plot for nausea. **b** Forest plot for vomiting (adverse effects of the blocks). RR relative risk, CI confidence interval, M–H Mantel–Haenszel, SD standard deviation, ESPB erector spinae plane block, SAPB serratus anterior plane block

Three articles [32, 33, 35] evaluated nausea and vomiting incidence. Both groups of patients reported similar incidences of nausea and vomiting (RR = 1.10, [0.66, 1.84], *p* = 0.72, *I*^2^ = 0%) (Fig. 10a) and RR = 1.27, [0.47, 3.44], *p* = 0.64, *I*^2^ = 0%) (Fig. 10b), respectively. No in-study heterogeneity was reported in either. An asymmetrical appearance on visual inspection of the funnel plots of both outcomes was seen (online supplemental appendix C).

## Discussion

This systematic review and meta-analysis aimed to compare the analgesic efficacy and safety profiles of the ESPB and SAPB in the context of breast surgery. While the primary analysis showed no significant difference in postoperative pain scores, ESPB demonstrated a statistically significant reduction in morphine consumption compared to SAPB. However, this difference did not reach clinical significance based on our predefined criteria [33]. Secondary outcomes also indicated no notable differences in pain scores and patient satisfaction between the two techniques.

The findings of this study hold implications for the management of postoperative pain in patients undergoing breast surgery since this is the first review conducted to compare the analgesic modalities in that population. This insight is crucial for clinicians in making informed decisions regarding pain management strategies, especially considering the increasing preference for minimally invasive and ultrasound-guided techniques in regional anesthesia. Future trials should assess analgesic efficacy over longer postoperative duration, i.e., 24 to 72 h. Additionally, among the included RCTs, only one study compared postoperative rehabilitation indicators among both groups [37]. Future studies with relatively large sample sizes comparing the efficacy in terms of postoperative rehabilitation indicators and the safety of blocks in terms of block-related complications and their effect on overall patients’ quality of life can also provide valuable evidence as to which block is superior to the other.

From an anatomical perspective, the ESPB and SAPB target different nerve distributions, which may explain their analgesic effects. The ESPB, a paraspinal fascial plane block, targets the dorsal and ventral rami of thoracic spinal nerves, providing a comprehensive sensory blockade that can extend to abdominal visceral analgesia. This broad range of analgesia may contribute to its efficacy in reducing postoperative morphine consumption. On the other hand, the SAPB selectively targets the lateral cutaneous branches of thoracic intercostal nerves, resulting in paresthesia across T2 to T9 dermatomes. This selective blockade is beneficial for managing pain localized to the surgical site, a common feature in breast surgery.

According to the Regional Anaesthesia UK, ESPB is one of the seven “Plan A” blocks for commonly encountered surgeries and acute pain. This is because ESPB can be administered at all the levels of the spine and provides analgesia to most body regions [39]. Moreover, the versatile use of ESPB especially in individuals on antithrombotic medications is due to its “superficial block” nature as opposed to deeper paravertebral or epidural blocks that may lead to bleeding due to the block administration [40]. The American Society of Regional Anesthesia has classified ESPB as a “low risk” for bleeding complications [41]. Given postoperative bleeding is a common and serious complication in breast surgery, the ESPB can be preferred [42]. The safety profile of ESPB is further exemplified with a lower incidence of pneumothorax when compared to other nerve blocks such as the paravertebral block [43]. Additionally, according to Luff et al. [44], trainee anesthetists have the most confidence in administering ESPB over other “Plan A” blocks and rates of block failures are less than 1/10th when performed by inexperienced anesthetists [45].

The SAPB can be injected superficially between the latissimus dorsi and serratus anterior muscle or deeply between the serratus anterior and intercostal muscle. With superficial SAPB providing more extensive effect and a greater safety profile than deep SAPB, there is discordance among the evidence since according to Moon et al. [46], the efficacy is similar while according to Piracha et al. [47], deep SAPB is more effective for postmastectomy pain control. More evidence can help decided the SAPB injection strategy. According to a meta-analysis by Meng et al. [48], SAPB can also reduce the incidence of chronic postsurgical pain after breast surgery highlighting its long-term use.

### Strengths and limitations

This systematic review, a comparison of ESPB and SAPB for postoperative analgesia following breast cancer surgery, is one in its own way and has not yet been the subject of a meta-analysis. It has several strengths, including adherence to reporting standards mentioned in PRISMA guidelines, a robust literature search strategy that included both the English and non-English RCTs, and a thorough assessment of the methodological quality and risk of bias of included studies. The inclusion of nine RCTs from diverse geographic regions enhances the generalizability of the findings. Moreover, the results were interpreted by taking into account MCID to avoid overestimating the statistically significant differences. Furthermore, the use of sensitivity and subgroup analyses helped address heterogeneity and provide a more precise understanding of the results.

However, the study also has limitations. The high degree of heterogeneity observed in some analyses suggests variability in study design, patient populations, and intervention protocols. Although sensitivity analyses partially addressed this issue, some residual heterogeneity remained unexplained that may have arisen from different local anesthetic agents and adjunct medications across studies. Lastly, while the overall strength of evidence was moderate to low for most outcomes, the limited number of studies and small sample sizes in some analyses hindered our ability to estimate some of the rare yet significant block-related complications. Despite these limitations, our study is the most up-to-date and comprehensive meta-analysis.

## Conclusion

Our review of nine RCTs revealed that patients undergoing breast cancer surgeries in ESPB group significantly have less postoperative opioid consumption and low demand of postoperative use of analgesia and took more time to use their first postoperative analgesia than those in SAPB group; however, this difference remained clinically unimportant. The postoperative pain scores, the incidence of nausea and vomiting, and the satisfaction score among both groups were comparable; hence, current evidence cannot define the relative superiority of one block over the other. Our findings warrant further research with standardized methodologies and a longer duration of analgesic efficacy assessment to yield robust evidence for better clinical applications.

## Supplementary Information

Supplementary Material 1.

Supplementary Material 2.

Supplementary Material 3.

## Data Availability

Data is provided within the manuscript or supplementary information files.
