# Supplementary material for: Analgesic efficacy and safety of erector spinae plane block versus serratus anterior plane block in breast surgery—a meta-analysis and systematic review of randomized controlled trials
Source: J Anesth Analg Crit Care. 2024 Dec 18;4:82. doi: 10.1186/s44158-024-00218-7 (PMC11657579; doi:10.1186/s44158-024-00218-7)
Supplement: Supplementary file 1 — Supplementary Material 1. [file 44158_2024_218_MOESM1_ESM.docx]

**ONLINE SUPPLEMENTARY APPENDIX A**

**Search Strategy Table**

| **Database** | **Search Strategy** | **Results** |
| --- | --- | --- |
| PubMed | (((((Erector spinae block) OR (Erector spinae plane block)) OR (ESPB)) OR (ESP)) AND (Postoperative analgesia)) AND ((((Breast cancer surgery) OR (Mastectomy)) OR (Modified Radical Mastectomy)) OR (MRM)) | 105 |
| Google Scholar | (Erector spinae block postoperative analgesia in Breast cancer surgery) | 2950 |
| Cochrane Library | (Erector spinae block):ti,ab,kw OR (Erector spinae plane block):ti,ab,kw OR (ESPB):ti,ab,kw AND (Postoperative analgesia):ti,ab,kw AND (Breast cancer surgery):ti,ab,kw" | 2137 |

MeSH: Medical education subject heading, ESBP: Erector spinae plane block, SAPB: Serratus anterior plane block
