# Supplementary material for: Analgesic efficacy and safety of erector spinae plane block versus serratus anterior plane block in breast surgery—a meta-analysis and systematic review of randomized controlled trials
Source: J Anesth Analg Crit Care. 2024 Dec 18;4:82. doi: 10.1186/s44158-024-00218-7 (PMC11657579; doi:10.1186/s44158-024-00218-7)
Supplement: Supplementary file 2 — Supplementary Material 2. [file 44158_2024_218_MOESM2_ESM.docx]

| Study (Year) | AUC Post operative pain score b/w 0-24hr static | | 24-hour Postoperative Oral morphine (mg) equivalent consumption | |
| --- | --- | --- | --- | --- |
|  | ESPB | SAPB | ESPB | SAPB |
| Eldemrdash 2019 [26] | 85.383 ± 95.652 | 122.25 ± 131.73 | 4 ± 2 | 7 ± 2 |
| Wang 2019 [28] | _ | _ | 48 ± 9.6 | 45.6 ± 7.2 |
| Elsabeeny 2020 [27] | 48.198 ± 1.359 | 45.231 ± 28.869 | 4.20 ± 1.64 | 4.00 ±1.55 |
| Shrivastava 2021 [34] | 54 ± 17.958 | 36 ± 17.958 | 1.54 ± 0.615 | 0.96±0.225 |
| Jiang 2021 [29] | _ | _ | 27.36 ±3.69 | 31.43 ± 1.88 |
| Sagar 2022 [33] | 59.55 ± 10.434 | 77.1 ± 9.075 | 185.00±36.635 | 227.50±47.226 |
| Ahuja 2022 [31] | 6.87 ± 22.62 | 8.46 ± 13.92 | 5.67 ± 0.84 | 7.52 ± 1.95 |
| Nyima 2023 [30] | 60.18 ± 17.958 | 88.2 ± 17.958 | 3.13 ± 1.44 | 4.33 ± 1.69 |
| Bedewy 2024 [32] | 115.0485 ± 42.4356 | 89.1273 ± 36.5715 | 4.6513 ± 2.4653 | 7.6546 ± 6.2631 |

**Table: Coprimary outcomes of the included studies**

ESPB: Erector spinae plane block, SAPB: Serratus anterior plane block; All values are represented in mean ± standard deviation.

| Study (year) |  | Eldemrdash  (2019) [26] | Wang (2019)  [28] | Elsabeeny  (2020)  [27] | Shrivastava (2021)  [34] | Jiang (2021)  [29] | Sagar (2022)  [33] | Ahuja (2022)  [31] | Nyima (2023)  [30] | Bedewy (2024)  [32] |
| --- | --- | --- | --- | --- | --- | --- | --- | --- | --- | --- |
| Pain score (static) at 0-hrs | E | 1.35 ± 2.35 | _ | 2 ± 1.01 | 2 ± 0.001 | _ | 0.35±0.489 | 2.33 + 4.42 | _ | 1.3598 ± 0.7978 |
|  | S | 1.35 ± 2.35 | _ | 1.011 ± 0.089 | 2 ± 0.001 | _ | 0.55±0.510 | 0.5 + 0.76 | _ | 2.1403 ± 1.5957 |
| Pain score (static) at 6-hr | E | 2.357 ± 2.358 | _ | 2 ± 0.001 | 2 ± 0.001 | 0.76 ± 0.6539) | _ | _ | _ | 2.0899 ± 1.3962 |
|  | S | 3 ± 3.14 | _ | 2 ± 1 | 2 ± 0.001 | 0.82 ± 0.8096 | _ | _ | _ | 2.3598 ± 0.7978 |
| Pain score (static) at 8hr | E | _ | 3.1±1.1 | _ | _ | _ | 1.70±0.657 | 0.64 + 0.76 | 1.88 ± 0.001 | _ |
|  | S | _ | 3.3±1.0 | _ | _ | _ | 2.35±0.745 | 0.64 + 0.76 | 2.38 ± 0.001 | _ |
| Pain score (static) at 12hr | E | 2.34 ± 2.65 | 3.3±1.1 | 1.011 ± 0.089 | 2 ± 0.001 | 1.24 ± 0.685 | 1.60±0.681 | 0.5 + 0.76 | 1.95 ± 0.001 | 4.3598 ± 1.9946 |
|  | S | 3.64 ± 3.93 | 3.3±1.1 | 1.011 ± 0.089 | 1 ± 0.001 | 1.28 ± 0.7161 | 2.25±0.550 | 0.64 + 0.76 | 2.805 ± 0.001 | 2.9281 ± 1.4361 |
| Pain score (static) at 24 hr | E | 3 ± 3.14 | 3.4±1.1 | 2 ± 1 | 1 ± 0.001 | 1.04 ± 0.6227 | 2.70±0.733 | 0.26 + 0.57 | 2.13 ± 0.001 | 2.6403 ± 0.7978 |
|  | S | 3.64 ± 3.93 | 3.4±1.1 | 2 ± 3 | 1 ± 0.001 | 1.16 ± 0.5916 | 2.65±0.745 | 0.64+0.76 | 2.76 ± 0.001 | 2.2302 ± 1.3962 |
| Pain score (dynamic) at 0 hr | E | _ | _ | _ | _ | _ | _ | 3.33 + 5.95 | _ | _ |
|  | S | _ | _ | _ | _ | _ | _ | 1.37 + 1.345 | _ | _ |
| Pain score (dynamic) at 8 hr | E | _ | 3.6±1.2 | _ | _ | _ | _ | 1.64 + 0.76 | 2.53 ± 0.001 | _ |
|  | S | _ | 3.8±1.2 | _ | _ | _ | _ | 1.64 + 0.76 | 3.25 ± 0.001 | _ |
| Pain score (dynamic) at 12hr | E | _ | 3.8±1.2 | _ | _ | 3.05 ± 0.7784 | _ | 1.5 + 0.76 | 2.72 ± 0.001 | _ |
|  | S | _ | 3.9±1.3 | _ | _ | 3.45 ± 0.9341 | _ | 1.354 + 0.76 | 3.32 ± 0.001 | _ |
| Pain score (dynamic) at 24 hr | E | _ | 3.9±1.3 | _ | _ | 1.325 ± 0.973 | _ | 1.26 + 0.57 | 2.92 ± 0.001 | _ |
|  | S | _ | 3.9±1.5 | _ | _ | 1.7 ± 0.9341 |  | 1.26 + 0.57 | 3.32 ± 0.001 | _ |
| Time to first analgesic request | E | 6.93 ± 1.13 | _ | 20.40 ± 4.98 | _ | 7.75 ± 0.76 | 6.875 ±0.7068 | 4.34 ± 0.98 | 9.57 ± 4.11 | _ |
|  | S | 5.72± 0.91 | _ | 19.00 ± 5.9 | _ | 5.79 ± 0.27 | 5.2167 ±0.7073 | 3.58±1.11 | 6.46 ± 2.95 | _ |
| Vomiting (%) | E | 5 (10%) | _ | _ | _ | 1 (3.33%) |  | 3 (7.5%) |  | _ |
|  | S | 2 (4%) | _ | _ | _ | 2 (6.66%) | _ | 3 (7.5%) | _ | _ |
| Nausea (%) | E | _ | 13 (26%) | _ | _ | 3 (10%) | _ | 8 (20%) | _ | _ |
|  | S | _ | 11 (22%) | _ | _ | 4 (13%) | _ | 7 (17.5%) | _ | _ |
| Time to first Postoperative Analgesic use (Hrs) | E | 6.93 ± 1.13 | _ | 20.40 ± 4.98 | _ | 7.75 ± 0.76 | 6.875±0.7068 | 4.34 ± 0.98 | 9.57 ± 4.11 | _ |
|  | S | 5.72± 0.91 | _ | 19.00 ± 5.9 | _ | 5.79 ± 0.27 | 5.2167 ±0.7073 | 3.58±1.11 | 6.46 ± 2.95 | _ |
| Patient satisfaction score | E | _ | _ | _ | _ | 8 ± 1.55 | _ | 5.7 ± 0.76 | _ | _ |
|  | S | _ | _ | _ | _ | 8 ± 1.55 | _ | 6.1 ± 0.87 | _ | _ |

**Table: Secondary outcomes of the included studies**

E: Erector spinae plane block, S: Serratus anterior plane block; Continuous outcomes are represented in mean ± standard deviation; Dichotomous outcomes are represented in RR (%).
