# Supplementary material for: Analgesic efficacy and safety of erector spinae plane block versus serratus anterior plane block in breast surgery—a meta-analysis and systematic review of randomized controlled trials
Source: J Anesth Analg Crit Care. 2024 Dec 18;4:82. doi: 10.1186/s44158-024-00218-7 (PMC11657579; doi:10.1186/s44158-024-00218-7)
Supplement: Supplementary file 3 — Supplementary Material 3. [file 44158_2024_218_MOESM3_ESM.docx]

**ONLINE SUPPLEMENTARY APPENDIX C**

**Funnel Plots of Co-Primary Outcomes and Secondary Outcomes**

| **AUC Pain scores (static) between 0-24 hours**  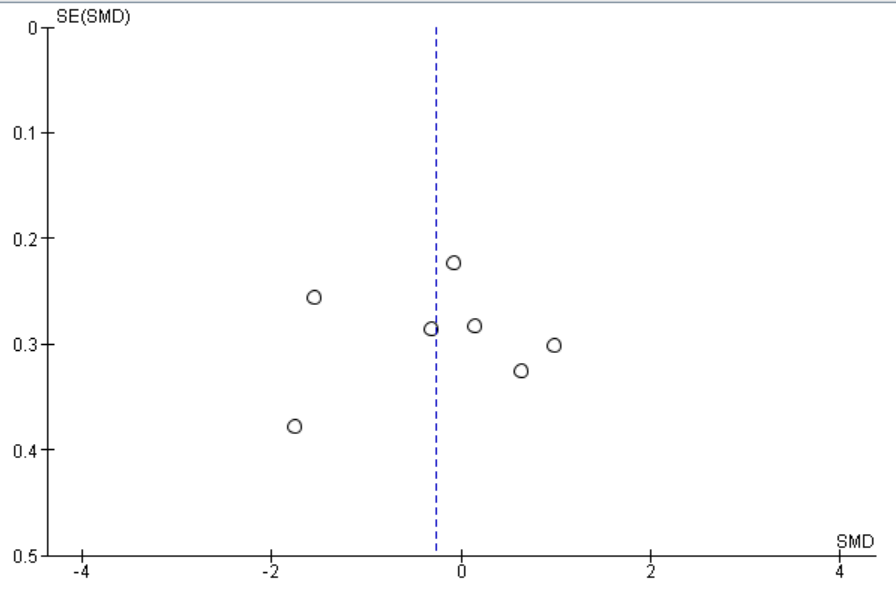 | **24-hour postoperative oral morphine (mg) equivalent consumption**  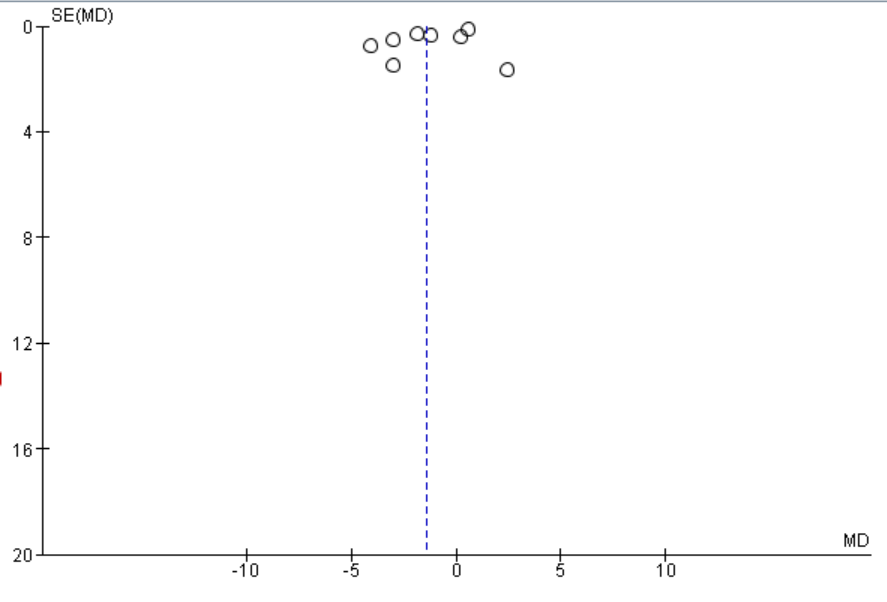 |
| --- | --- |
| **Pain scores (static) at 0-hour**  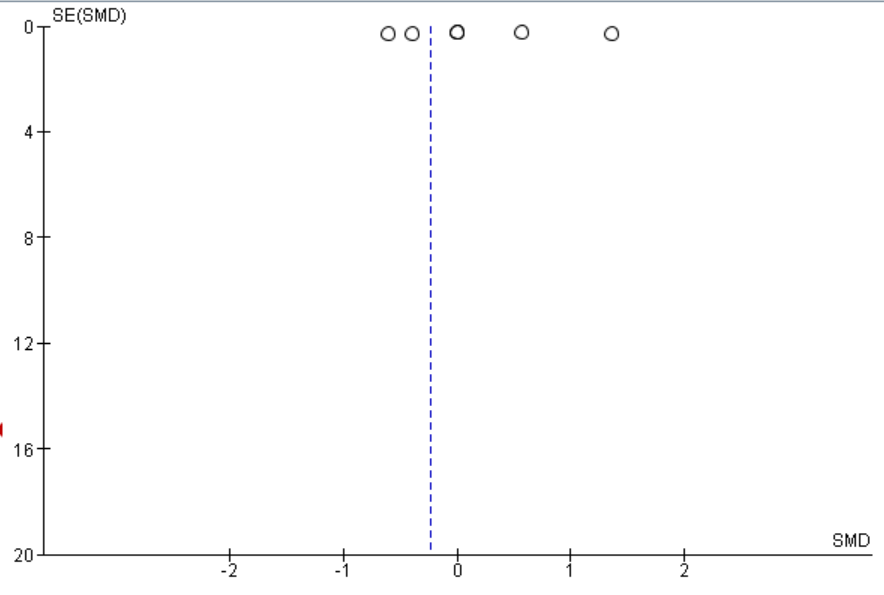 | **Pain scores (dynamic) at 0 hours**  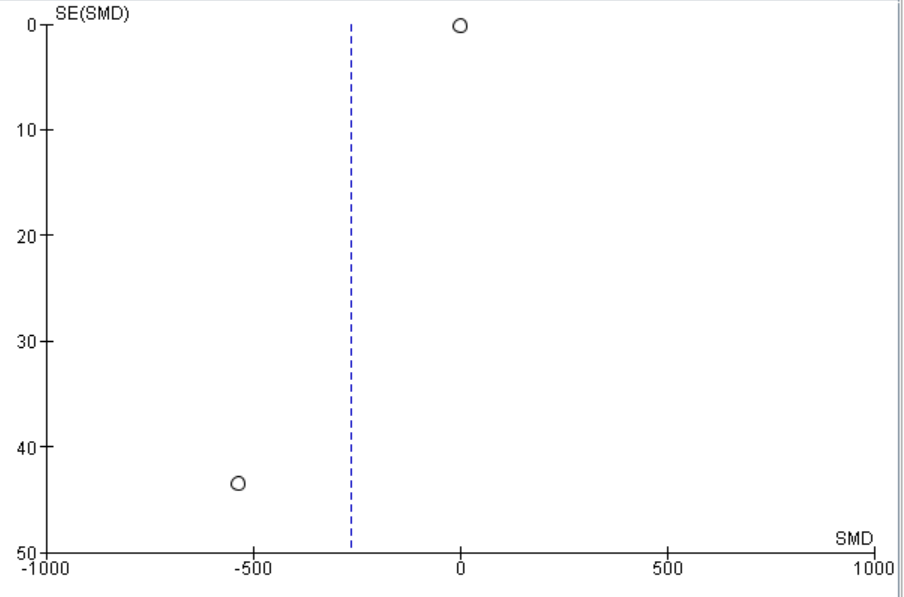 |
| **Pain scores (static) at 8-hours**  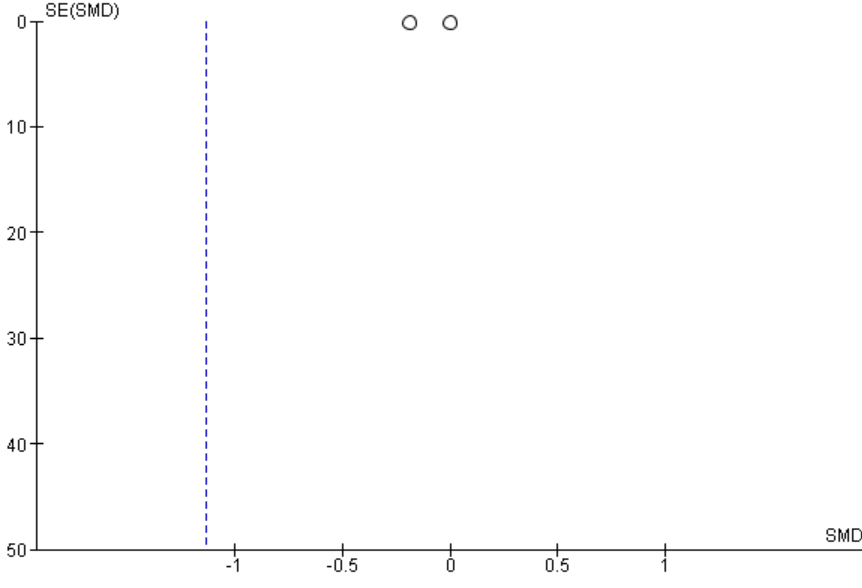 | **Pain scores (dynamic) at 8-hours**  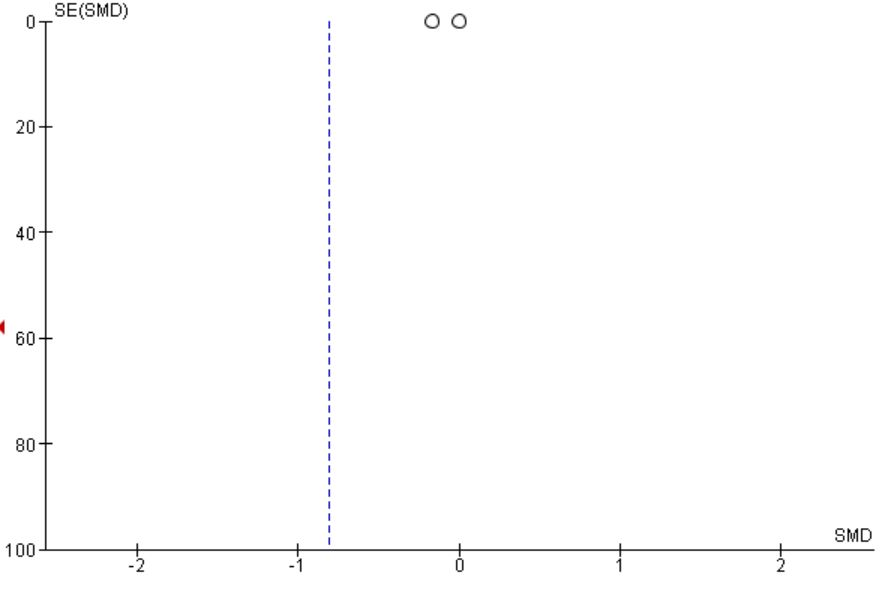 |
| **Pain scores (static) at 12-hours**  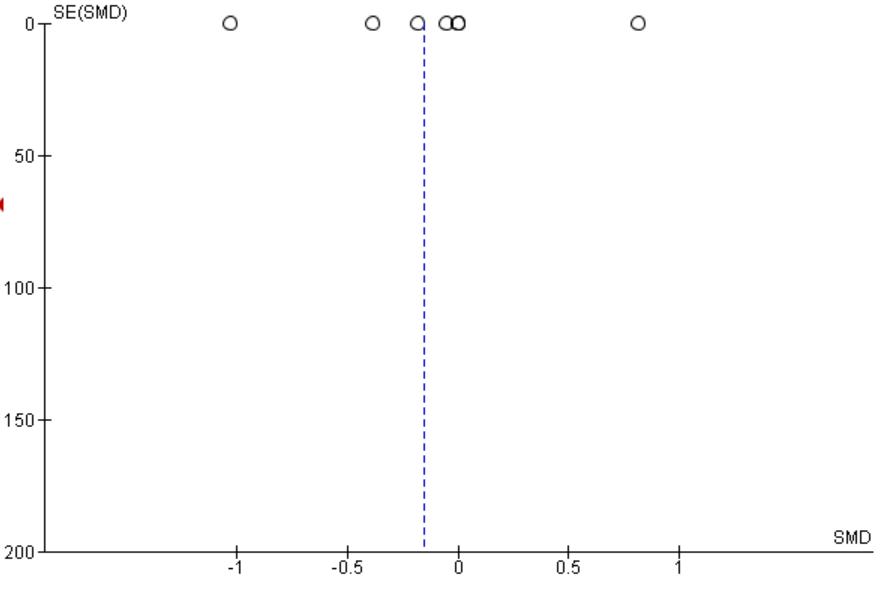 | **Pain scores (dynamic) at 12-hours**  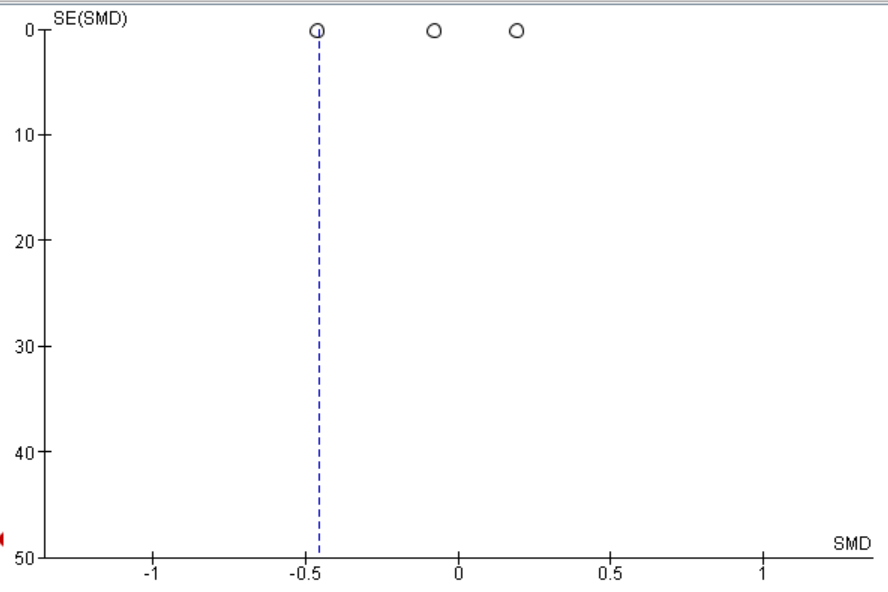 |
| **Pain scores (static) at 24-hours**  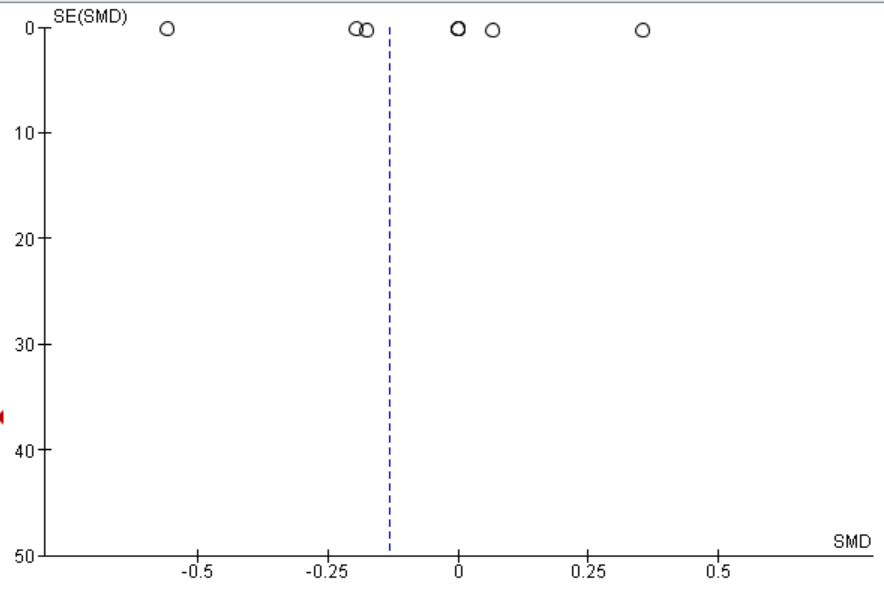 | **Pain scores (dynamic) at 24-hours**  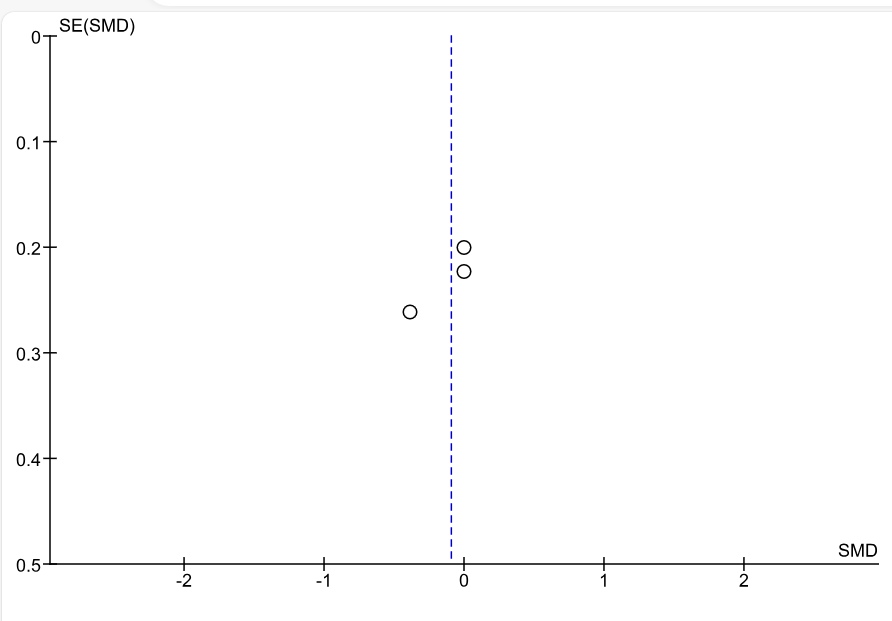 |
| **Pain scores (static) at 6-hour**  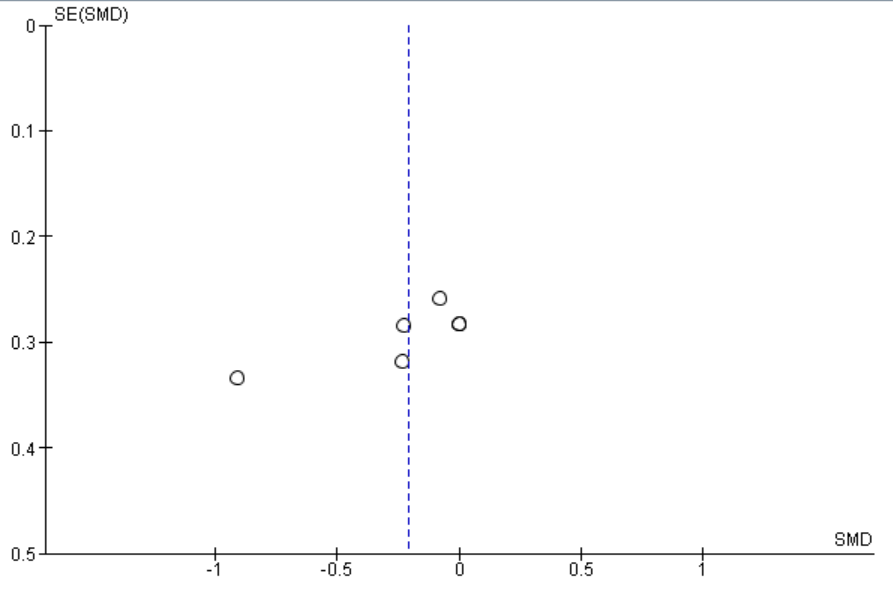 | **Time to request the first dose of postoperative analgesia.**  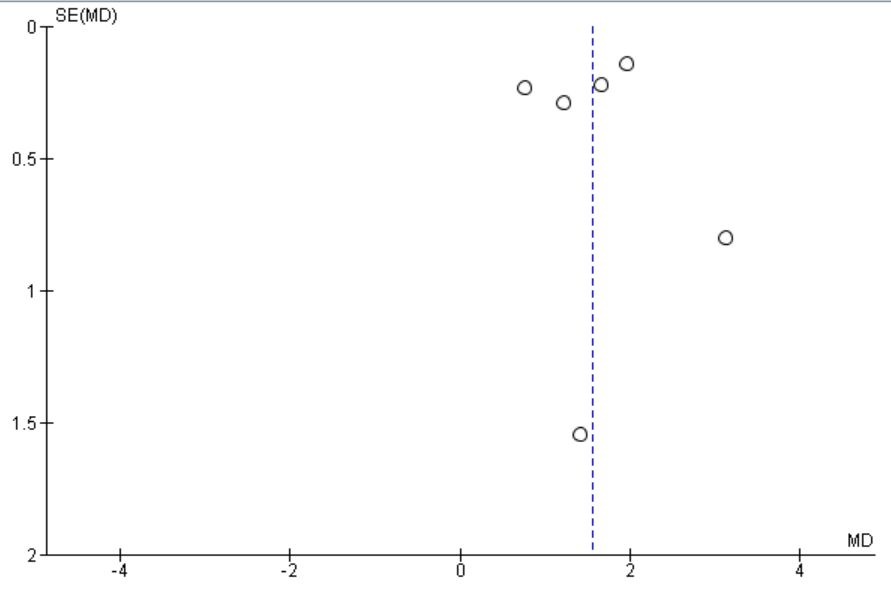 |
| **No. of patients requested postoperative analgesia**  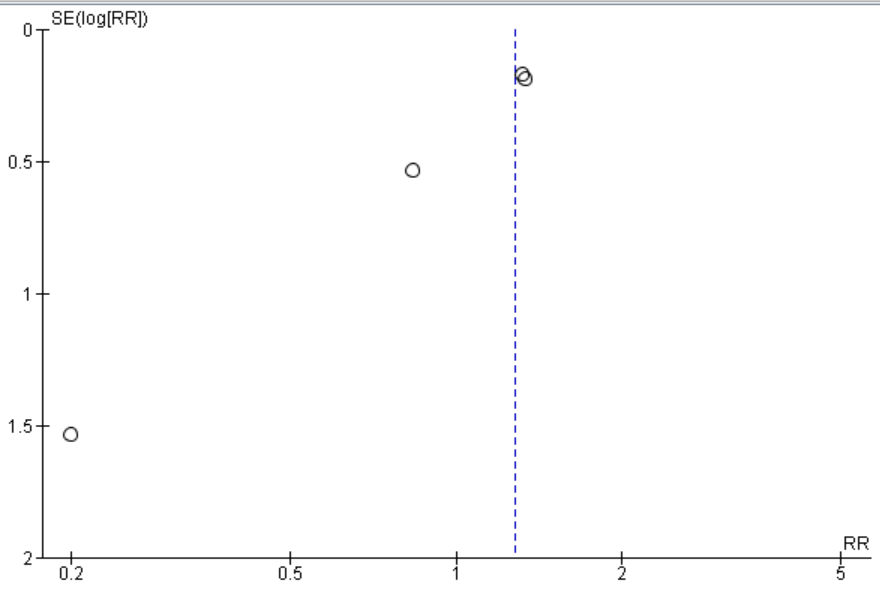 | **Satisfaction Score**  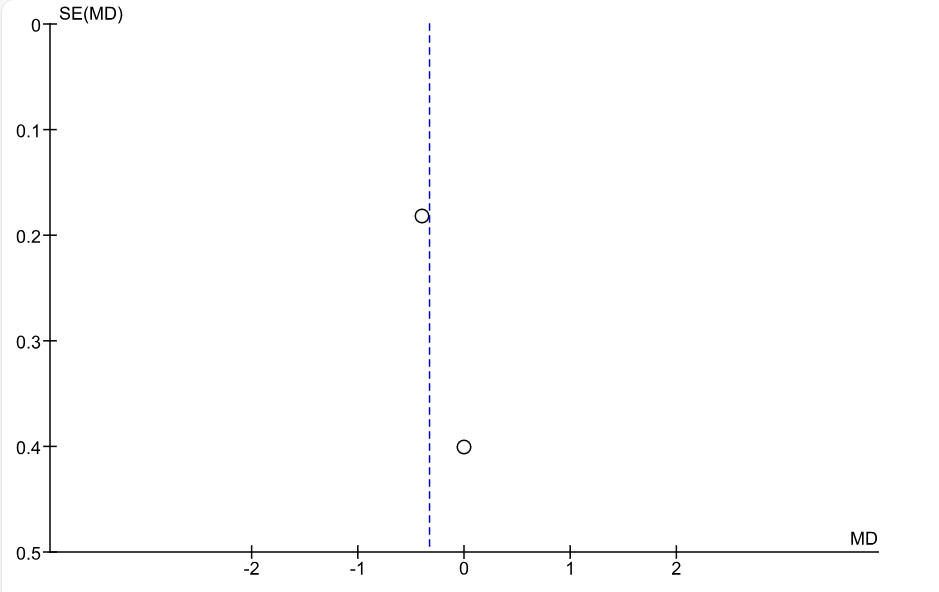 |
| **Postoperative nausea**  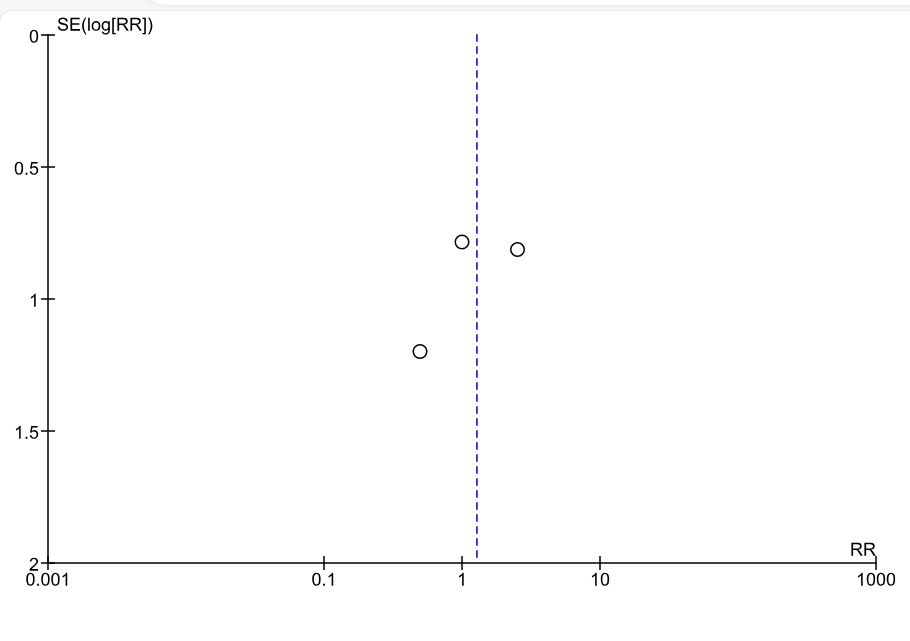 | **Postoperative vomiting**  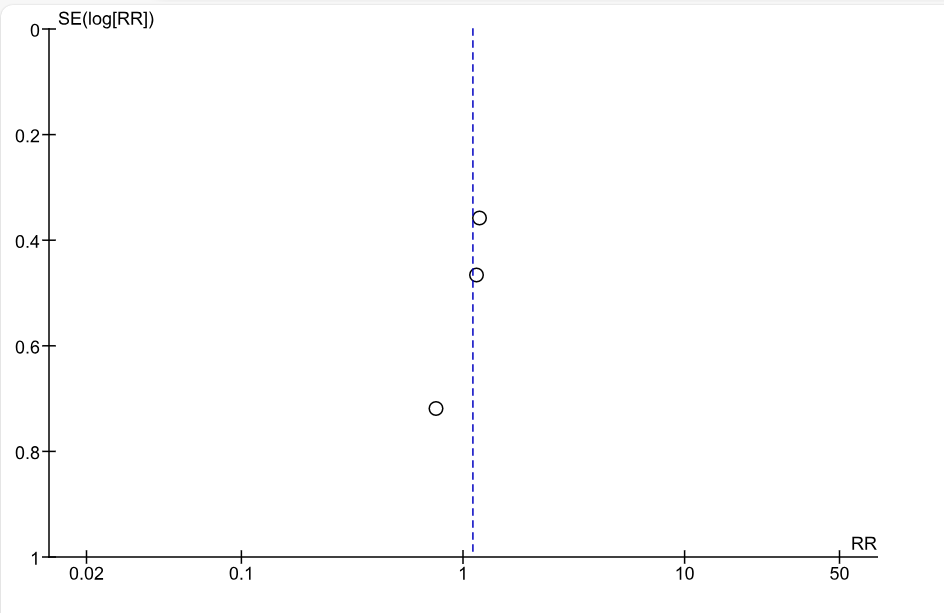 |
